# Selective Sweeps in a Nutshell: The Genomic Footprint of Rapid Insecticide Resistance Evolution in the Almond Agroecosystem

**DOI:** 10.1093/gbe/evaa234

**Published:** 2020-11-04

**Authors:** Bernarda Calla, Mark Demkovich, Joel P Siegel, João Paulo Gomes Viana, Kim K O Walden, Hugh M Robertson, May R Berenbaum

**Affiliations:** 1 Department of Entomology, University of Illinois at Urbana-Champaign; 2 United States Department of Agriculture, Agricultural Research Service, Commodity Protection and Quality Research, Parlier, California; 3 Crop Sciences Department, University of Illinois at Urbana-Champaign

**Keywords:** pesticide, population genomics, convergent evolution, nucleotide diversity, evolutionary rescue

## Abstract

Among the most familiar forms of human-driven evolution on ecological time scales is the rapid acquisition of resistance to pesticides by insects. Since the widespread adoption of synthetic organic insecticides in the mid-twentieth century, over 500 arthropod species have evolved resistance to at least one insecticide. Efforts to determine the genetic bases of insecticide resistance have historically focused on individual loci, but the availability of genomic tools has facilitated the screening of genome-wide characteristics. We resequenced three contemporary populations of the navel orangeworm (*Amyelois transitella*), the principal pest of almond orchards in California, differing in bifenthrin resistance status to examine insecticide-induced changes in the population genomic landscape of this species. We detected an exceptionally large region with virtually no polymorphisms, extending to up to 1.3 Mb in the resistant population. This selective sweep includes genes associated with pyrethroid and DDT resistance, including a cytochrome P450 gene cluster and the gene encoding the voltage-gated sodium channel *para*. Moreover, the sequence along the sweep is nearly identical in the genome assembled from a population founded in 1966, suggesting that the foundation for insecticide resistance may date back a half-century, when California’s Central Valley experienced massive area-wide applications of DDT for pest control.

**Significance**

Although it is widely known that insecticide resistance is rapidly acquired by target insects, comprehensive population genomic scans that assess the evolutionary trajectory of the development of resistance are scant. This is particularly true for insect pests that are nonmodel organisms, some of which are especially important due to the substantial damage they can cause to crops and as consequence to food supply. This study looks at the genome footprints and consequences of intensive insecticide usage in a pest of almonds in California. We detected a large selective sweep, an unequivocal signature of positive selection, in a region of the insect genome comprising several genes with potential roles in target insensitivity and insecticide detoxification.

## Introduction

Among the most familiar forms of human-driven evolution on ecological time scales is the rapid acquisition of resistance to pesticides in agriculture (Hendry et al. 2017). Compared with selection exerted by most natural mortality factors, pesticide selection is generally extremely strong and specific, and resistance can develop exceptionally fast (Hawkins et al. 2019). Since the widespread adoption of synthetic organic insecticides in the mid-twentieth century, more than 500 arthropod species, over half of which are agricultural pests, are known to have evolved resistance to at least one insecticide (Whalon et al. 2008; Sparks and Nauen 2015). Resistance evolution remains an enduring challenge to the use of synthetic insecticides in agriculture and human disease vector control. Historically, efforts to determine the genetic and evolutionary bases of insecticide resistance have focused on allelic variation and gene expression changes at individual loci associated with metabolic resistance or target-site insensitivity. At the genomic and population scale, studies of selection for insecticide resistance have focused mainly on drosophilid model species (Steele et al. 2015; Schmidt et al. 2017; Duneau et al. 2018) or medically important mosquito species (Fouet et al. 2017; Kamdem et al. 2017; Kotsakiozi et al. 2017). Surprisingly few genome-wide population studies of field-acquired insecticide resistance have been carried out on agricultural pests, and little is known about the evolutionary origins and dynamics that govern this process (Oakeshott et al. 2003; Pélissié et al. 2018).

By querying the full genome and its variation among individuals and among populations, population genomic approaches can illuminate multiple specific targets of positive selection, as well as whole affected regions. A genome-wide approach can also help to distinguish this positive selection from the effect of the demographic history of the populations under examination (Akey et al. 2004). A classic hallmark of positive selection is a reduction in nucleotide diversity in genome regions flanking genes that have been the targets of such selection (Kaplan et al. 1989). This shift in allele frequencies, or “selective sweep” (Kim and Stephan 2002), depends on recombination rates in the genomic region and on the timing and strength of the underlying selective pressure. Classic selective sweeps, or “hard sweeps,” where heterozygosity reaches near-zero values at or very close to the causative mutation, are rare in nature and are not readily reproducible in laboratory settings (i.e., experimental evolution studies) which often result in soft sweeps (Burke 2012; Messer and Petrov 2013).

Production of almonds (*Prunus dulcis*), a multibillion-dollar industry centered in the California Central Valley, can be severely affected by the navel orangeworm (*Amyelois transitella*, Lepidoptera: Pyralidae), the most important pest of this commodity. Insecticides are routinely and intensively used in almond orchards to manage this pest. Although insecticides approved for use include representatives from five structural classes, pyrethroids have been widely used in recent years due to their low cost and high efficacy. Prior to 2013, insecticide resistance was not known to occur in navel orangeworm populations; in 2013, however, 10-fold resistance to bifenthrin was documented in populations in Kern County (Demkovich et al. 2015). A reference genome for *A. transitella* was assembled in 2013 (https://i5k.nal.usda.gov, last accesed November 1, 2020). This reference was based on the “SPIRL-1966” line established in the USDA laboratory facility in the San Joaquin Valley in 1966, during a period preceding the use of pyrethroids and during which the Central Valley was subjected via crop-dusting to “thousands of tons of DDT alone” (Cory et al. 1971). DDT shares the same metabolic target as pyrethroids and was widely used from 1945 until 1972. Early use of DDT was associated to preadaptation for resistance to pyrethroid insecticides in several mosquito populations (Rongsriyam and Busvine 1975; Chadwick et al. 1977; Hemingway et al. 1989).

In this study, we took advantage of the availability of the *A. transitella* reference genome and of the detailed record of past insecticide usage in the California Central Valley to interrogate the origins and evolutionary trajectory of insecticide resistance in this pest at the genomic level. We evaluated nucleotide diversity and genomic differentiation in three modern, geographically separate *A. transitella* populations with different histories of bifenthrin use and levels of bifenthrin resistance. We also assessed gene expression changes in resistant and susceptible genotypes. We demonstrate the existence of a large selective sweep which includes, among others, genes encoding for cytochrome P450s and the *para* gene, the target of pyrethroids and DDT.

## Results

### Nucleotide Diversity and Tajima’s *D*

**Fig. 1:**
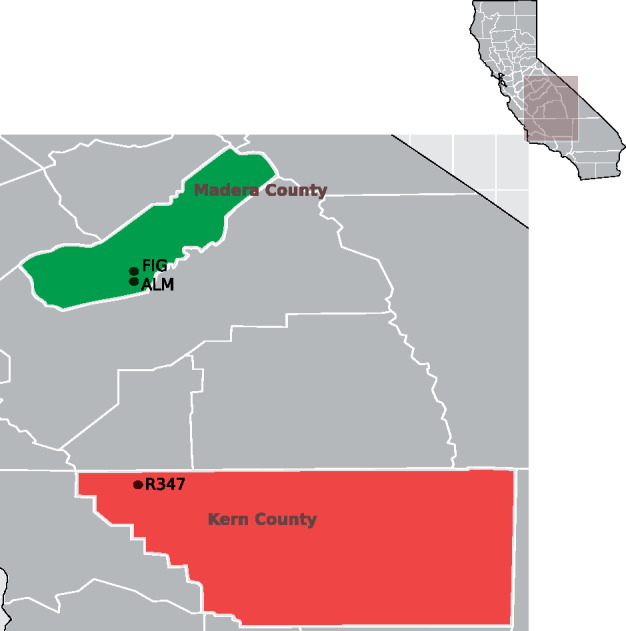
Sampling locations in the California Central Valley. Three populations of the navel orange worm were sampled. “ALM” and “FIG” were sampled in Madera County. The “R347” population was sampled in Kern County.

**Fig. 2:**
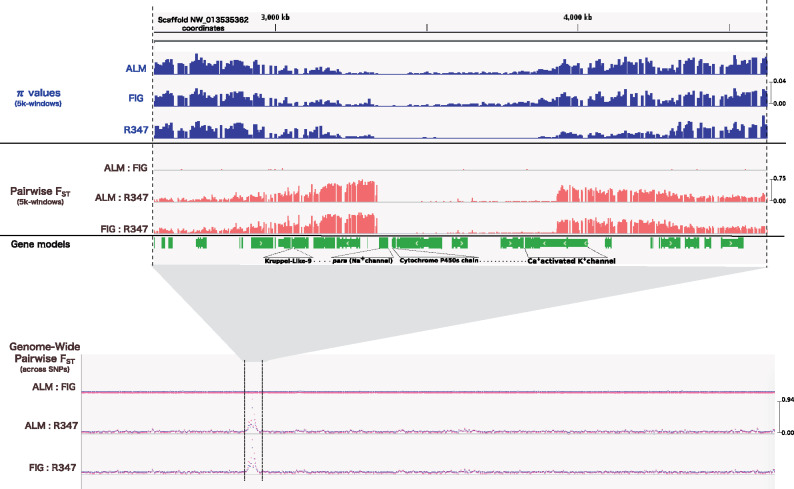
Selective sweep region in scaffold NW_013535362.1 of the *Amyelois transitella* genome. Top to bottom: Top panel shows π values in the sweep and its flanking regions calculated across 5-kb windows in bifenthrin-susceptible (ALM and FIG), and resistant (R347) populations. Middle panel shows pairwise *F*_ST_ estimates calculated in the sweep and its flanking regions across 5-kb windows for the three populations pairs. Bottom panel shows the “genome-wide” pairwise *F*_ST_ values calculated across SNPs, red and blue overlapped lines are results from Popoolation2 (Kofler, Pandey, et al. 2011) and “Poolfstat” v.1.0.0 R package https://cran.r-project.org/web/packages/poolfstat, respectively.

Three populations of *A. transitella* originating from Madera County (ALM and FIG), and Kern County (R347) in the California Central Valley were sampled and sequenced (fig. 1). Of these three populations, only the R347 displays resistance to bifenthrin. The sequencing resulted in roughly 82× raw coverage of the 409-Mb *A. transitella* genome for each of the populations sequenced (supplementary table S1, Supplementary Material online). The reads were mapped to our reference genome (7,301 scaffolds with N50 = 1,586,980, L50 = 62, and ∼36× coverage) and SNPs were called for each of the populations (table 1). Nucleotide diversity was calculated with the π statistics and the deviation of variability from null expectations under a neutral model with Tajima’s *D*, both metrics were calculated across nonoverlapping 5-kb-long windows for each of the populations (table 1). To identify regions of low nucleotide diversity (low π), we sorted the 5-kb regions according to their calculated π values. The lowest π values were compared with those across the genome and with averages in nearby regions. In addition, the windows with the lowest π values were assessed for their calculated Tajima’s *D* values at the 90% CI in the same region. With this analysis, an unusually large region displaying reduced genetic diversity across the three populations was readily detected (fig. 2). This region showed π values as low as zero in the R347 population and ranging from 0.00002 to 0.00041 in ALM and FIG, a drop from 0.04022 in the flanking regions within the same scaffold and from a genome-wide average of 0.17911 for R347 and 0.01752 and 0.01799 for ALM and FIG, respectively. We narrowed down this region of low nucleotide diversity to the region starting ∼2.92 Mb in scaffold NW_013535362.1 and extending up to base position 3.8 Mb in the ALM and FIG populations to encompass 38 genes, and up to base position 4.32 Mb in the R347 population to encompass 43 genes (fig. 2).

**Table 1:** Number of SNPs, Nucleotide Diversity (π), and Tajima’s *D* Values Calculated on 5-kb Nonoverlapping Windows across the Whole Genome and in the NW_013535362.1 Scaffold in Three Populations of *Amyelois transitella*: ALM and FIG Susceptible, R347 Resistant

	ALM	FIG	R347
Number of SNPs	10,592,326	10,303,426	9,704,346
π (genome-wide average)	0.17911	0.017517	0.017089
Minimum value of π (scaffold)	0.000019 (NW_013535362)	0.000047 (NW_013535362)	0.00000 (NW_013535362)
Maximum value of π (scaffold)	0.039661 (NW_013535371.1)	0.0400582 (NW_013535371.1)	0.039509 (NW_013535951.1)
Tajima’s *D* (genome-wide average)	0.04528	0.03524	0.2424
**In NW_013535362.1 only (calculated on coding regions)**			
π average	0.0148181	0.0142969	0.0131416

**Fig. 3:**
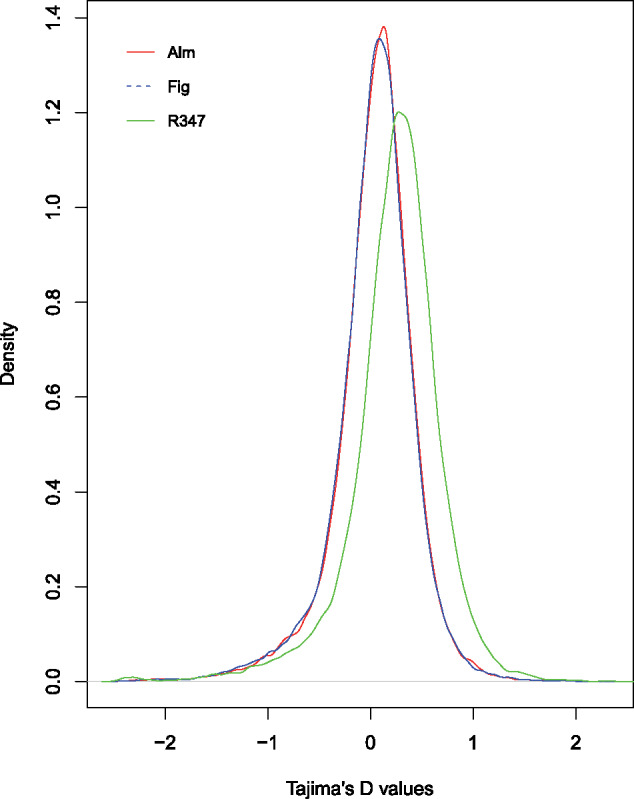
Distribution of Tajima’s *D* values across three populations of *Amyelois transitella*. Values were calculated on 5-kb non-overlapping windows across the full genome. The curves show heavy tails on both positive and negative values. Negative Tajima’s *D* suggests an excess of low-frequency mutations that could be due to population expansion, background selection, or selective sweeps, whereas positive Tajima’s *D* indicates balancing selection.

The distribution of Tajima’s *D* values for 5-kb windows for each of the three populations (fig. 3) shows heavy tails at both positive and negative sides of the range. The resistant genotype (R347) displayed higher genome-wide average Tajima’s *D* (between 0.239 and 0.246, CI = 90%). compared with ALM and FIG (between 0.0417 to 0.0487 and 0.0316 to 0.038, respectively, at the same confidence level) (table 1). Negative Tajima’s *D* indicates an excess of low-frequency mutations that could be due to population expansion, background selection, or selective sweeps, whereas positive Tajima’s *D* indicates balancing selection (the maintenance of alternative alleles that benefit the population). The top 1% and bottom 1% values of the Tajima’s *D*, which fall in the extreme positive and negative part of the range, respectively, were used to evaluate regions departing from neutrality (supplementary table S2, Supplementary Material online). The lowest Tajima’s *D* values range from −2.428 to −2.5016 in the three populations, and were found in the same region where the selective sweep was detected, confirming that this region of low polymorphism is indeed a region that was positively selected and which significantly departs from a neutral model of natural selection. Unexpectedly, an examination of the read alignments to the reference population SPIRL-1966 shows that the nucleotide sequence of the reference is nearly identical to that of the three other populations along the hard sweep region. The reference genome sequence is also nearly identical to that of R347 in the regions flanking the selective sweep (supplementary fig. S1, Supplementary Material online).

To further narrow down the origin of the selective sweep, the π metric was recalculated on a gene-by-gene basis on the predicted gene models across the entire NW_013535362 scaffold. Of the 190 annotated gene models, 170 had enough sequencing coverage to calculate nucleotide diversity at the set cut-offs described in the Materials and Methods section. In the ALM population, nucleotide diversity reached zero (π = 0) at the *protein kish-A* gene (XM_013328236.1), with no SNPs detected along this coding sequence, followed by the voltage-gated sodium channel *para* (XM_013328250.1), with a single SNP. In the FIG population, the lowest π value (0.0001) was also found in *para*, followed by the gene encoding the cytochrome P450 CYP6B56 (XM_013328369.1). In the R347 population, π was zero at the gene encoding CYP6B56 (table 2). The coverage and number of SNPs for each of the genes in the scaffold are detailed in supplementary table S3, Supplementary Material online. There are approximately 15 genes in the flanking region to the left of the hard-sweep, where polymorphisms start to accumulate in all three populations, including cytochrome c oxidase, a cyclin-dependent kinase, a phosphomevalonate kinase, and *Krüppel-like-9* transcription factor, among others. To the right of the sweep, where only R347 exhibits low nucleotide diversity, we found a small conductance calcium-activated potassium channel (SkCa2), two uncharacterized proteins, and an extensin-like protein-coding gene (table 2).

**Table 2:** Nucleotide Diversity Estimates (π) for Genes in the Sweep Region (Scaffold NW_01353536)

Gene_ID	Start	End	Gene Name	π		
				ALM	FIG	R347
XM_013328221.1	2922570	2976883	Dynein heavy chain	0.0152	0.0137	0.0135
XM_013328219.1	2977372	2991119	ADAM 17-like protease	0.0113	0.0090	0.0094
XM_013328243.1	3002489	3003978	Cytochrome c oxidase	0.0066	0.0057	0.0045
XM_013328367.1	3012239	3045757	Tyrosine-protein kinase	0.0076	0.0065	0.0068
XM_013328167.1	3044389	3048122	Uncharacterized	0.0025	0.0014	0.0038
XM_013328206.1	3049409	3053897	DET1 homolog	0.0054	0.0036	0.0104
XM_013328222.1	3055117	3067241	Cyclin-dependent kinase 7	0.0041	0.0032	na
XM_013328168.1	3067370	3078948	Tubulin polyglutamylase TTLL13-like	0.0070	0.0056	0.0121
XM_013328280.1	3097871	3099727	GPI mannosyltransferase	0.0012	0.0008	0.0018
XM_013328281.1	3101631	3107700	Methyl-CpG-binding domain protein	0.0068	0.0052	0.0120
XM_013328283.1	3128643	3157699	Phospholipid-transporting ATPase	0.0065	0.0055	0.0137
XM_013328423.1	3159383	3164900	Uncharacterized	0.0015	0.0014	0.0012
XM_013328361.1	3164948	3193977	Unconventional myosin-like	0.0059	0.0050	0.0050
XM_013328247.1	3197609	3199024	Krüppel-like factor 9	0.0050	0.0042	0.0035
XM_013328326.1	3204704	3279423	Protein groucho	0.0026	0.0026	0.0026
XM_013328229.1	3304902	3307354	Phosphomevalonate kinase	0.0022	0.0025	0.0017
XM_013328250.1	3344559	3350148	Sodium channel protein *para*	0.0000	0.0001	0.0001
XM_013328169.1	3387986	3390408	CYP6B54	0.0004	0.0008	0.0004
XM_013328169.1	3393988	3396394	CYP6B55	0.0004	0.0008	0.0004
XM_013328369.1	3399243	3401314	CYP6B56	0.0001	0.0001	0.0000
XM_013328341.1	3403909	3420607	Protein ariadne	0.0001	0.0003	0.0001
XM_013328377.1	3421473	3423241	Uncharacterized	0.0001	0.0002	0.0002
XM_013328378.1	3423369	3430390	Isoleucine–tRNA ligase	0.0002	0.0007	0.0003
XM_013328269.1	3433176	3489393	dmX-like protein 2	0.0012	0.0014	0.0003
XM_013328231.1	3490744	3524106	Endophilin-A%2C transcript variant X1	0.0014	0.0011	0.0005
XM_013328234.1	3525167	3528547	Polyubiquitin-C	0.0030	0.0028	0.0021
XM_013328235.1	3529722	3548710	Ras GTPase-activating	0.0016	0.0020	0.0004
XM_013328236.1	3549071	3550177	Protein kish-A	0.0000	0.0005	0.0001
XM_013328238.1	3550487	3554188	Protein prenyltransferase alpha subunit	0.0008	0.0013	0.0002
XM_013328171.1	3584697	3630468	Uncharacterized	0.0019	0.0028	0.0003
XM_013328188.1	3631722	3638019	Uncharacterized	0.0016	0.0022	0.0006
XM_013328187.1	3745239	3796518	Protein unc-13 homolog A	0.0021	0.0024	0.0002
XM_013328228.1	3800374	3817393	Actin-binding Rho-activating protein-like	0.0025	0.0038	0.0002
XM_013328172.1	3826528	3849030	Gonadotropin-releasing hormone II receptor	0.0026	0.0043	0.0005
XM_013328189.1	3852565	4034421	Small conductance Ca-activated K channel	0.0088	0.0096	0.0036
XM_013328392.1	4090436	4108229	Uncharacterized	0.0071	0.0068	0.0026
XM_013328198.1	4240437	4246388	Extensin-like	0.0151	0.0137	0.0076
XM_013328173.1	4247890	4251407	Uncharacterized	0.0056	0.0051	0.0038
XM_013328174.1	4268658	4270209	Uncharacterized LOC106129578	0.0078	0.0066	0.0053
XM_013328217.1	4270710	4272681	Tyrosine–tRNA ligase%2C cytoplasmic	0.0022	0.0025	0.0007
XM_013328325.1	4275218	4323747	RNA polymerase II elongation factor ELL	0.0062	0.0053	0.0046

Note.—Only genes with read coverage (>50) were used to calculate π are shown (41 out of 44 genes). Dark gray cells π > 0.02, light gray and white cells π < 0.002, and white cells are defined hard sweep region in all three populations.

### Mutations in the Hard Sweep Region

We manually screened the few polymorphisms segregating along the region where all three populations have near-zero π values. Only two genes had nonsynonymous and nonconservative mutations in all or in a portion of the reads covering the position across the three populations (supplementary table S4, Supplementary Material online). One of these was the *para* gene (which also showed π values equal to zero), with the mutation L934F. All three populations carried the mutation in 100% of the sequenced reads covering the position, in contrast with the reference genome (supplementary fig. S2*A*, Supplementary Material online). This mutation corresponds to the known *kdr* (knock down resistance) mutation in the *para* gene that confers target-site resistance to DDT and pyrethroids (Kalra et al. 1967; Farnham 1977; Williamson et al. 1996). Although the SPIRL-1966 reference genome shows nearly identical nucleotide sequence along *para*, it does not have the *kdr* mutation. In the absence of population-level data for the reference genome, we confirmed that this population did not carry the *kdr* mutation by resequencing the region flanking this mutation on ten SPIRL-1966 individuals collected in 2012 and preserved in our laboratory (supplementary fig. S3, Supplementary Material online). A second gene encoding a *Krüppel-like-9* factor (KL-9) had nonsilent mutations segregating in 82% and 91% of the sequenced reads from ALM and FIG, respectively, and in only 9% of the reads in the resistant R347. KL-9 overlaps the region where all three populations have low diversity and the region where only the R347 population has reduced π values. The read alignment to these two genes and all of the polymorphisms segregating on each of the three populations across the genomic sequences are shown in supplementary figure S2*B*, Supplementary Material online.

### Population Differentiation (*F*_ST_) and Principal Component Analysis

Genetic differentiation measured as *F*_ST_ between populations showed that ALM and FIG are probably indistinguishable as separate populations (*F*_ST_ −0.004, as negative *F*_ST_ values are effectively zero). The *F*_ST_ between ALM and R347 was 0.0289 and between FIG and R347 was 0.0290. These values are highly reflective of the demographic origins of each population and agree with the genome-wide average Tajima’s *D* calculated earlier. Genes controlling traits that differ between populations might present large differences in allele frequencies, thereby generating higher *F*_ST_ values comparable with average *F*_ST_ across the genome (Myles et al. 2007). We scanned for outlier loci showing SNPs with large differences in allele frequency by calculating *F*_ST_ on a by-SNP basis utilizing two different methods (see Materials and Methods). The SNPs with top 1% *F*_ST_ values across the whole genome were concentrated in the regions flanking the identified selective sweep (fig. 2). These high *F*_ST_ values were all found in pairwise comparisons with the resistant genotype (i.e., ALM vs. R347 or FIG vs. R347). *F*_ST_ were recalculated after eliminating the sweep region from the genome, and the resulting *F*_ST_ values were almost identical to the ones obtained with the full genome, indicating that, other than the region surrounding the selective sweep, these populations are also differentiated due to geographically restricted selection that is not related to insecticide selection and could include population bottlenecks, genetic drift, or migration.

**Fig. 4:**
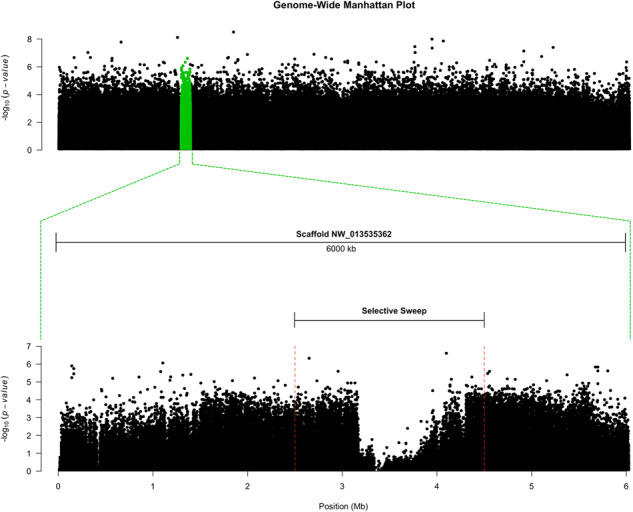
Manhattan plot for the tree tested populations of *Amyelois transitella*. The plot is based on the *P* values of Mahalanobis distances between calculated *z* scores derived from regressing each SNP by 2 principal components (*k* = 2). For details, see Materials and Methods. Highly differentiated SNPs were screened based of FDR-corrected *P* values (supplementary table S5, Supplementary Material online).

The *F*_ST_ method may not account for unexpected population structure and requires that each sequenced individual is assigned to a single population, an assumption that might not be valid. To verify our findings by overcoming the limitations of *F*_ST_, we utilized an approach based on principal component analysis (PCA) on the calculated major allele frequencies. With this test, we explored the 20 most-differentiated SNPs according to their FDR-corrected *P* values. Of those, only 2 SNPs were differentiated between the resistant and susceptible populations: one in scaffold NW_013535509.1, in an intergenic region near an ecdysteroid kinase gene cluster, and a second in the NW_013535362.1 scaffold, just downstream of the selective sweep in position ∼4.1 Mb (fig. 4 and supplementary table S5, Supplementary Material online). The PCA calculated for the major allele frequencies also reflected the population demographics (supplementary fig. S4, Supplementary Material online).

### Cytochrome P450s, Gene Expression Analyses, and d*N*/d*S* Rates Ratios

**Fig. 5:**
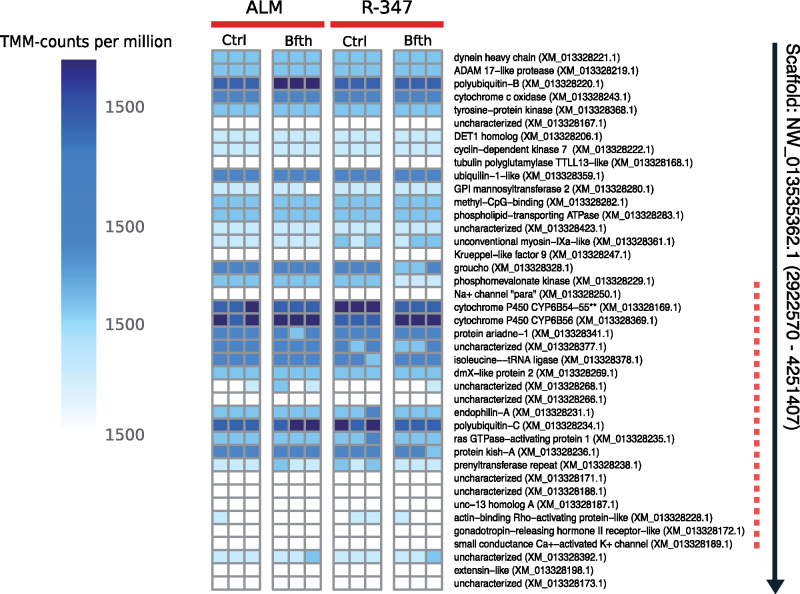
RNA-seq results showing the transcript-per-million-mapped-reads (TMM) normalized read counts in the NW_013535362.1 scaffold of the *Amyelois transitella* reference assembly. ALM, susceptible population; R347, resistant population; Ctrl, control diet without insecticide; Bfth, diet with bifenthrin. Black arrow in the left shows the scaffold and its direction. Dotted red line in the left shows the region of extreme low nucleotide diversity. Note that CYP6B54 and CYP6B55 are predicted in the genome as a joint transcript (missing a stop codon), for that reason, the read counts are merged for both, and we could not draw any conclusion regarding transcript levels for these two genes.

To further probe the genes in the sweep region and surrounding areas, we carried out RNA-seq with midguts of ALM and R347 larvae that consumed bifenthrin or a control diet. We inspected the expression values of the (a priori selected) 42 genes that fall within the defined selective sweep. Of these, the CYP6B tandem showed the highest expression values across all the treatments (up to 15 times higher than the average FPKM in the sweep region, and up to 17 times more than the genome-wide average). In addition, a pattern consistent with elevated expression of CYP6B56 upon bifenthrin treatment in the R347 was supported with ANOVA tests (*F* = 6.8, *P *≤* *0.05) (fig. 5). A gene coding for polyubiquitin-C also exhibited increased transcript levels upon bifenthrin exposure in the susceptible ALM population, but this difference was not significant at the set threshold (fig. 5 and supplementary table S6, Supplementary Material online). For CYP6B54 and CYP6B55, we could not draw conclusions as the predicted gene model in the reference genome is missing a stop codon and the reads are counted jointly for both transcripts. To obtain additional confirmation of the findings of the RNA-seq analysis and to differentiate CYP6B54 from CYP6B55, we conducted a qPCR experiment with the same samples by designing unique primers specific for each of the three P450s in the cluster. The transcript coding for CYP6B54 showed constitutively higher expression in R347 compared with ALM, independent of the bifenthrin treatment (*t*-test = 2.12, *P* < 0.05) (supplementary fig. S5, Supplementary Material online); CYP6B55 did not show statistically significant differences between treatments or between populations and CYP6B56 failed to amplify. Because there is only a limited range of nucleotide sequence that we could use to design primers that will not cross-amplify these three closely related genes, we could not repeat the test for CY6B56. An additional transcript coding for the Krüppel-like transcription factor 9 was added to the test, and it showed low expression across samples and treatments in agreement with the RNA-seq data.

The analysis of nonsynonymous to synonymous substitutions rate ratios (ω) in CYP6B-coding sequences from seven species of Lepidoptera showed that a model allowing for an increase in the rate of positive selection along the branches originating after the duplication of the most recent common ancestor of the CYP6B tandem (Branch No. 24 supplementary fig. S6, Supplementary Material online) explained the data better than a null hypothesis model of no change in selection pressure across the whole CYP6B phylogeny (*X*^2^ = 45.52, *P*_corr_* *≤* *0.000) (same ω in all branches of the tree), further supporting a role of these P450s in adaptation. We also found significant differences in selective pressure after the duplication that led to CYP6B54 and CYP6B55 (Branch No. 23) (*X*^2^ = 28.6, *P*_corr_* *≤* *0.000) but not to CYP6B56 (Branch No. 25) (supplementary fig. S6, Supplementary Material online).

### Bioassays and Analysis of Historical Data on Insecticide Usage

To determine the functional significance of the identified genomic signatures, we compared levels of resistance to bifenthrin and DDT in ALM and R347 with CPQ, a population derived from the original SPIRL-1996 line that lacks the *kdr* mutation. The CPQ population was the most susceptible to bifenthrin, with an LC_50_ value of 0.38 ppm; in contrast, the R347 population displayed the highest level of resistance, with a LC_50_ value of 24.27 ppm, confirming field observations of insecticide failure. The LC50 of ALM was 7.45, comparable to that of FIG reported by Bagchi et al. (2016). There was no difference in LC_50_ for DDT between the ALM and the R347 populations, both displaying relatively high resistance to DDT (LC_50_ = 259.85 and 310.33 ppm, respectively) compared with CPQ (LC_50_ = 25.32 ppm) (table 3). These values are suggestive of a direct association between the presence of *kdr* and DDT resistance but not between *kdr* and bifenthrin resistance. Statewide in California, according to data from the California Department of Pesticide Regulation, we found that the use of bifenthrin in pounds increased 3.5- to 10.1-fold, and by area 4.4- to 5.2-fold from 2007 to 2013. Applications per pound of product across that period were consistently larger in Kern County than in Madera County (supplementary fig. S7 and table S7, Supplementary Material online).

**Table 3:** Comparison of Toxicity of Bifenthrin and DDT in Four Strains of the Navel Orangeworm (*Amyelois transitella*) with the Presence or Absence of the “Knock-Down Resistance” (*kdr*) Mutation in the *para* Gene

Population	Bifenthrin 48 h LC_50_ (95% CI)	DDT 48 h LC_50_ (95% CI)
ALM *(kdr+)*	7.45 (5.90–9.64)	259.85 (216.71–326.0)
FIG *(kdr+)*	7.20 (5.35–11.08)^a^	—
R347*(kdr+)*	24.27 (18.19–33.09)	310.33 (249.37–424.29)
CPQ *(kdr−)*	0.38 (0.31–0.46)^a^	25.32 (19.73–30.84)

Note.—LC_50_, median lethal concentration; CI, confidence intervals; ALM, FIG, R347, and CPQ, populations of *A. transitella* used in the study.

a LC_50_ identified in Bagchi et al. (2016).

## Discussion

Among the identifiable footprints of positive selection are selective sweeps. Footprints associated with evolved insecticide resistance could help understanding processes and events that ultimately shape the genomes of insects in response to human-driven selection. In this study, we utilized pooled DNA sequencing (Pool-seq) as an efficient and cost-effective method (Schlötterer et al. 2014) to screen the genomes of three spatially distinct populations of the navel orangeworm, one of which recently evolved resistance to bifenthrin. We confirmed the existence of a large selective sweep with virtually no polymorphisms across a 0.5-Mb-long region in all these modern populations. Values of Tajima’s *D* in this same region are below −2, a strong indication of a departure from neutrality. In addition, the regions flanking both sides of the selective sweep show very high *F*_ST_ values between the resistant R347 and either of the susceptible populations. This difference could be due to differences in the point in time when the frequency of the sweep increased or when mutations accumulated after the sweep reached fixation, but they could also be due to the presence of an allele or alleles possibly linked to the difference in resistance in either or both of these regions.

### Insecticide Resistance and the Selective Sweep

Reports of selective sweeps of the size identified in this study are vanishingly rare. Tian et al. (2009) reported a 1.1-Mb sweep in domesticated *Zea mays*, with similar evidence of multiple selection targets; this sweep likely resulted artificial selection for domestication exerted over thousands of years (Kistler et al. 2018). In humans, a >1.5-Mb-long hard sweep surrounding the lactase (*LCT*) gene in lactose-tolerant northern European populations (Bersaglieri et al. 2004) and in African pastoralist populations (Tishkoff et al. 2007), a textbook example of both selective sweeps and convergent evolution, is considered one of the strongest selection signatures so far reported in the human genome (Tishkoff et al. 2007). As in the case of the *Z. mays* selective sweep, the LCT-associated selective sweep emerged over the past ∼7,000 years coincident with the domestication of cows.

Within the context of insecticide selection, only a handful of studies have identified sweeps associated with resistance and, in most cases to date, the sweeps were in relatively small regions (<200 kb). Several of these studies identified genes encoding cytochrome P450 associated with the sweeps. Schlenke and Begun (2004), reported a ∼100-kb selective sweep associated with a *Doc* transposable element inserted upstream of *Cyp6g1* in *Drosophila simulans*, but this sweep was only marginally associated with DDT resistance*.* Soft, incomplete selective sweeps were found in the α-esterase gene cluster that contains the polymorphic *LcαE7* gene encoding forms of the protein conferring organophosphate insecticide resistance in the Australian sheep blow fly *Lucilia cuprina* (Rose et al. 2011). A region containing the quantitative trait loci responsible for pyrethroid resistance in the malaria vector mosquito *Anopheles funestus* displayed characteristics of a selective sweep, which was then narrowed to the *CYP6P9A* and *CYP6P9B* P450 genes tandem (Barnes et al. 2017). Song et al. (2015) analyzed nine Z chromosome-linked loci in different populations of the Old World bollworm *Helicoverpa armigera* and detected a region possibly indicative of a selective sweep surrounding the *Cyp303a1* locus. Among studies reporting selective sweeps associated with insecticide resistance, the one with findings most similar to ours is the study of Kamdem et al. (2017), who compared urban and rural populations of mosquitoes in the *Anopheles gambiae* complex in Cameroon, where DDT is still used for malaria control, and identified a selective sweep containing circa 80 genes inclusive of the *kdr* locus.

### The Para Gene

The *kdr* locus is a single recessive point mutation resulting in an amino acid substitution from L to F in the S6 transmembrane segment of domain II of the *para* gene, a voltage-gated sodium channel, and the main target of both pyrethroids and DDT (Soderlund and Bloomquist 1989). The *kdr* locus has long been associated with resistance to DDT and to other neurotoxic insecticides, including pyrethrins and pyrethroids in many insect species (Kalra et al. 1967; Farnham 1977; Miyazaki et al. 1996; Pittendrigh et al. 1997; Bass et al. 2007; Haddi et al. 2012; Dong et al. 2014). There are thus, limited ways in which *para* as a target of neurotoxic insecticides can evolve resistance, providing an example of “biochemically precise” (Hartley et al. 2006) convergent evolution. Similar cases of convergency were observed for the alpha-esterase coding genes that evolved resistance to organophophate insecticides in several insect species (Hartley et al. 2006; ffrench-Constant 2007), and in Na, K-ATPase coding genes in disparate insect species that evolved resistance to plant-derived cardenolides (Dobler et al. 2012).

In this study, we found that nucleotide diversity in the *para* gene reaches zero or near-zero values in the three *A. trasnsitella* populations, and the *kdr* mutation is present in ALM, FIG, and R347 populations and absent in the reference genome and in the CPQ population (sequenced only for the *kdr* locus). According to our bioassays, resistance to DDT coincides with the presence of the *kdr* mutation, but resistance to bifenthrin does not, indicating that other genes or loci are also responsible for the R347 resistance. It is of special interest that the *kdr* mutation has been linked to preadaptative resistance to pyrethroids in multiple mosquito species (Rongsriyam and Busvine 1975; Chadwick et al. 1977).

### Cytochrome P450s

Assays with piperonyl butoxide, a P450 synergist, have implicated cytochrome P450s in pyrethroid detoxification in *A. transitella* (Demkovich et al. 2015). Of the genes in the sweep that could be involved in insecticide resistance, there is a cluster of three cytochrome P450 genes (*CYP6B54*, *CYP6B55*, and *CYP6B56*), where *CYP6B56* has a π value of zero in R347. Although the precise substrate specificity of these *A. transitella* P450s has not been defined, they belong to a CYP subfamily for which there is both direct (Li et al. 2004) and indirect (Wang and Hobbs 1995; Ranasinghe and Hobbs 1998; Zhang et al. 2010; Qiu et al. 2012) evidence of involvement in pyrethroid resistance.

Tandem clusters of P450s are likely recent duplications that have persisted due to their adaptive value (Ohno 1970; Nei and Rooney 2005). The existence of P450s with the ability to detoxify phytochemicals likely served as a preadaptation for rapid evolution of resistance to insecticides, particularly those originally derived from or inspired by phytochemicals (Li et al. 2007; Omura 2013). Gene expression analyses showed increased levels of CYP6B56 transcripts in susceptible and resistant populations exposed to bifenthrin compared with control, and a comparison of nonsynonymous to synonymous rates of amino acid substitution indicates increased positive selection along the branches originating after the most recent common ancestor of the three CYP6Bs in the tandem.

### Other Genes in the Sweep and Possible Regulatory Regions

Genes in the flanking region to the left of the hard-sweep, where polymorphisms start to accumulate in all three populations and where population differentiation (*F*_ST_) is high between resistant and susceptible populations, include a cytochrome c oxidase, an enzyme that has been directly associated with pyrethroid resistance in at least one insect species (Pridgeon and Liu 2003). In addition, within this flanking region is the *KL9* transcription factor, which has been implicated in regulation of P450 expression in mammals (Koh et al. 2014), and which showed segregating nonsilent mutations that differ between the resistant and both susceptible populations. To the right of the sweep, where only R347 exhibits low nucleotide diversity, we identified a gene encoding a small conductance calcium-activated potassium channel (SkCa2). Although calcium and chloride ion channels are known to interact with pyrethroids in mammals (Soderlund 2012), to date there is no evidence that insect SkCa2 channels are targeted by pyrethroids or that they are involved in resistance. In addition, PCA analyses identified two highly differentiated SNPs between susceptible and resistant populations, one of which is located just downstream of the selective sweep in the intron–exon boundary of a functionally uncharacterized locus. The other highly differentiated SNP was identified near a cluster of three ecdysteroid kinase coding genes (EcKLs). Evidence exists that EcKLs could be responsible detoxification in insects by direct or indirect phosphorylation of xenobiotics (Scanlan et al. 2020), but our transcriptomic data did not provide evidence of EcKLs constitutive transcript enrichment in the resistant line or of induction of any EcKL transcripts in either genotype upon application of bifenthrin.

### Limitations

The ability of Pool-seq data to infer population allele frequencies relies heavily on sample size. We used 100 individuals from each population to ascertain our ability to infer signatures of selection. With that sample size Pool-seq outperform other techniques that rely on full genome resequencing or marker-based sequencing such as RAD-seq (Schlötterer et al. 2014). Pool-seq, however, has some limitations, the most important of which is the loss of haplotype information, which is critical to infer additional population genomic parameters.

In addition, because the economic impacts of *A. transitella* as a pest species is geographically circumscribed, the demand for the development of genomic research tools to facilitate research on this nonmodel species has been limited, so there are no available genetic maps or calculated recombination rates for this species, which limits our ability to infer the coalescence by simulations. Access to museum species would have also improved the study and facilitated the interpretations.

Based on our findings, a plausible scenario to account for the presence of the large hard sweep in contemporary field populations of *A. transitella* is a stacking of selective forces that prevailed at a time preceding the founding of the SPIRL-1966 colony and that persisted under continuous selection until a new reinforcing selective pressure arrived in the form of pyrethroids. DDT, which shares the same metabolic target as pyrethroids, was widely used from 1945 until 1972, when virtually all federal registrations for its use in the United States were canceled by the Environmental Protection Agency. Despite its curtailed use, DDT and its derivatives are known to remain in the environment, persisting in the soil as contaminants and moving from there across trophic levels and geographic regions (Mansouri et al. 2017). SPIRL-1966 was founded with individuals collected during an era of massive environmental exposure to DDT; although individuals from a laboratory colony in 2016 did not carry the *kdr* mutation, our findings are consistent with a loss of the mutation after four decades of relaxed selection under laboratory conditions (Hanai et al. 2018). Alternatively, the SPIRL-1966 never carried the *kdr* mutation, in which case one or more of the cytochrome P450s present in the sweep are the main candidate targets of selection and causative of the sweep. The persistence of resistance alleles (including *kdr* and *Rdl* that confer resistance to dieldrin) after decades of discontinuation of the insecticides that selected for them has been documented in mosquito species (Schechtman and Souza 2015; Grau-Bové et al. 2020). Taken together, our findings provide an illustration of the ability of humans, in efforts to manage economically important pests, to alter the pace of evolution and the genomic composition of species over relatively short periods (Palumbi 2001).

## Materials and Methods

### Sampling and Resequencing

One hundred individuals from each of three navel orangeworm populations displaying different levels of bifenthrin resistance in the Central Valley were sampled and sequenced in pools. The “ALM” and “FIG” populations of *A. transitella* were collected from almonds and fig orchards in Madera County as larvae (J.P.S., USDA-ARS, Parlier, CA) in 2016. Larvae from the bifenthrin-resistant population “R347” were collected from almond orchards in Kern County and sent to us by Brad Higbee (Trécé) in 2016. All three populations were maintained in an incubator at the University of Illinois at Urbana-Champaign at temperatures of 28 ± 4 °C and photoperiod of 16:8 (L:D) h cycle until individuals eclosed, whereupon they were frozen at −80 °C. Genomic DNA was extracted and prepared into sequencing libraries following standard procedures as described in supplementary methods, Supplementary Material online. Libraries were paired-end sequenced for 150-base-long reads on one lane of the Illumina HiSeq 4000 (Illumina, San Diego, CA).

### Alignment, SNP Calling, and Identification of Genomic Regions under Positive Selection

Reads were trimmed for residual adapters and quality using Trimmomatic v. 0.32 (Bolger et al. 2014) with the following parameters: PE (for paired-end reads), ILLUMINACLIP: Truseq3_Nextera_PE.fa (adapters file), and MINLEN: 50 (minimum length to keep the read). The trimmed reads were aligned to the *A. transitella* reference genome (NCBI accession ASM118610v1) using bwa mem v. 0.7.12-r1039 with paired-end mode (Li 2013). The resulting SAM files were sorted and optical- and PCR-derived sequencing duplicates were marked and removed using Picard v.1.48 (Broad Institute, http://broadinstitute.github.io/picard/, last accesed November 1, 2020). Low-quality alignments (mapping quality score <20), improper pairs, and unmapped mate reads were removed using SAMtools v.1.7 (Li et al. 2009). A pileup file was created for each separate library using the mpileup command from SAMtools. Indels were identified and separated from the main pileup files. The three pileup libraries were then subsampled for uniform SNP coverage of reads using “identify-genomic-indel-regions.pl” and “subsample-pileup.pl” scripts from the Popoolation v.1.2.2 package with the following parameters: –min-qual (minimum base quality) = 20, –target-coverage = 50, and –max-coverage (the maximum allowed coverage) = 100 (Kofler, Orozco-terWengel, et al. 2011). The subsampling was carried out to minimize biases in population genomic metrics affected by sequencing errors and copy number variation that could skew coverage in affected regions.

Nucleotide diversity (π), and Tajima’s *D* were calculated for each of the populations using 5-kb-long nonoverlapping windows with the “Variance-sliding.pl” script from Popoolation. The Tajima’s *D* values were used to build an empirically derived distribution using 10,000 bootstrap replications with sampling with replacement with R v3.5.1. Confidence intervals of the mean (CI) were calculated on Tajima’s *D* values. On a separate pipeline, a multiple-pileup file was created that incorporated the three trimmed and quality-filtered mapped libraries, indels were removed as described, and libraries were synchronized using “mpileup2sync.jar” from Pooplation2 software (v.1.201) (Kofler, Pandey, et al. 2011). The 3-populations pileup file was subsampled for uniform coverage and pairwise *F*_ST_ values were calculated using the “fst-sliding.pl” script from Popoolation2 on a per-SNP basis.

For pooled DNA sequencing, *F*_ST_ values might present bias if the sample is too small or if nonequimolar amounts of DNA for each of the individuals from each population were used. Bias can also be introduced during the PCR amplification step before sequencing (Cutler and Jensen 2010). Even though *F*_ST_ calculation in Popoolation2 implements a bias correction, the estimates are still deemed biased according to Hivert et al. (2018). For that reason, to validate our *F*_ST_ estimates, the mpileup synchronized file obtained from Pooplation2, was converted to a pooldata object for the “Poolfstat” v.1.0.0 R package (https://cran.r-project.org/web/packages/poolfstat). Both methods yielded similar results, but Popoolation2 does not report overall *F*_ST_ between populations; accordingly, we report results from both packages. “Pcadapt” v.1.1 (Luu et al. 2017) was used to detect outlier SNPs based on PCA. In PCAdapt, *z* scores were calculated based on the original set of SNPs with *K* = 2. Outliers were identified on the *z* scores vector using Mahalanobis distances. The distances were transformed into *P* values assuming a chi-squared distribution with K degrees of freedom (Luu et al. 2017).

### Insecticide Bioassays

To establish the median-lethal concentrations (LC_50_) for bifenthrin and DDT in the sequenced populations, we conducted feeding assays with semisynthetic artificial diet (Waldbauer et al. 1984). Bifenthrin (Chem Service Inc., West Chester, PA), or DDT (Sigma-Aldrich Co., St. Louis, MO) was stirred into the diet at different concentrations for each population and poured into separate 1-oz (28 ml) cups to set. Treatments and concentrations were: bifenthrin in methanol—ALM: 2 ppm, 5 ppm, 10 ppm, 12 ppm, 15 ppm, 24 ppm; DDT—ALM: 50 ppm, 100 ppm, 200 ppm, 300 ppm, 400 ppm; bifenthrin—R347: 8 ppm, 16 ppm, 24 ppm, 48 ppm, 75 ppm; DDT—R347: 50 ppm, 100 ppm, 200 ppm, 300 ppm, 400 ppm; DDT—CPQ: 10 ppm, 20 ppm, 35 ppm, 50 ppm, 75 ppm, 100 ppm. Four neonates were transferred with a soft brush into each plastic cup containing bifenthrin or methanol as the solvent control. Twenty larvae from each population were exposed to their respective bifenthrin or DDT concentrations and each assay was replicated three times per concentration. Neonate mortality on diets was assessed after 48 h and scored according to a movement response after being touched by a soft brush. Probit analysis (SPSS version 22, SPSS Inc., Chicago, IL) was applied to identify median-lethal concentrations (LC_50_). Differences between populations were considered significant if their respective 95% confidence intervals in the Probit analysis did not overlap. We were unable to perform these assays with the FIG population because we did not establish a colony from the population used for sequencing; however, for the purposes of comparison, we incorporated the bifenthrin LC_50_ established in our laboratory for this population by Bagchi et al. (2016).

### RNA-Seq Analyses

We used RNA extracted from midguts from *A. transitella* samples from a concurrent study (in review). Briefly, fifth instar caterpillars from the ALM and the R347 populations were placed on artificial diet containing either 0.5 ppm bifenthrin or methanol as the control solvent. After 48 h, the larval midguts were dissected and flash-frozen in liquid nitrogen. There were three replicates per treatment and population combinations for a total of 12 samples, each consisting of a single midgut. Total RNA from each of the 12 samples was extracted using a Nucleospin RNA kit (Macherey-Nagel, Düren, Germany) according to the manufacturer’s protocol. The RNA was sequenced on an Illumina HiSeq 4000 (Illumina, San Diego, CA). After sequencing, reads were preprocessed, mapped to the reference genome, and quantified for differential expression following previously described methods (Calla et al. 2017; supplementary methods, Supplementary Material online). The data were deposited in the NCBI SRA repository under accession number PRJNA548705.

### Quantitative Real-Time PCR

About 1 µg of each of the RNA samples that were used for RNA-seq were reverse transcribed to cDNA with a Protoscript II kit (New England Biolab, Ipswich, MA). The resulting cDNA was diluted 10-fold with nuclease-free water. qPCR reactions were assembled with SYBR green fast-qPCR mix (Thermo Fisher Scientific, Walthasm, MA) and primers specific for either *CYP6B541*, *CYP6B55*, *CYP6B56*, or *Krüppel-like factor (KL-9)*. The *GADPH* (Glyceraldehyde 3-phosphate dehydrogenase) was used as housekeeping gene. Primer sequences are in supplementary table S8, Supplementary Material online.

### Molecular Evolution (d*N*/d*S*) Analyses

Nonsynonymous to synonymous substitution rate ratios (ω) were calculated across the phylogeny of a representative set of 7 species of Lepidoptera with fully curated sets of cytochrome P450s, for a total of 22 CYP6B sequences. Nucleotide coding sequences were codon aligned using MUSCLE (Edgar 2004), and the alignment was trimmed from the ends to remove misaligned regions. A maximum-likelihood gene tree was constructed with RaXML (Stamatakis 2014), and a species tree was used to create a gene-species tree reconciliation with Notung (Chen et al. 2000). The tree with the most parsimonious topology and the codon aligned nucleotide sequences were used to run the CodeML software from PAML v.4.8 (Yang 1996, 2007), applying branch models. Detailed methods for each model, trees, and statistics are in supplementary methods, Supplementary Material online.

### Sequencing the Para Locus in Laboratory Populations

DNA was extracted from ten frozen whole-body fifth instar larvae from the reference genome population SPIRL-1966, and ten midguts of fifth instar larvae of the CPQ population that fed on semisynthetic artificial diet (Waldbauer et al. 1984). Extractions were carried out using an E.Z.N.A. insect DNA kit (Omega Bio-Tek, Norcross, GA) according to manufacturer’s instructions. Primers were designed using Primer3 (Untergasser et al. 2012) as implemented in Geneious software (v 9.1.8) (Kearse et al. 2012) to sequence the region of the *para* gene containing the *kdr* mutation (Forward 5′-ACCAAGGTGGAACTTCACAGAT-3′ Reverse 5′-AGCAATTTCAAGAAGTCAGCAACA-3′). Amplicons were Sanger sequenced and trace analysis and alignments were performed in Geneious.

## Supplementary Material

Supplementary data are available at *Genome Biology and Evolution* online.

## Supplementary Material

evaa234_Supplementary_Data
