# Supplementary material for: Selective Sweeps in a Nutshell: The Genomic Footprint of Rapid Insecticide Resistance Evolution in the Almond Agroecosystem
Source: Genome Biol Evol. 2020 Nov 4;13(1):evaa234. doi: 10.1093/gbe/evaa234 (PMC7850051; doi:10.1093/gbe/evaa234)
Supplement: evaa234_Supplementary_Data [file evaa234_supplementary_data.zip › Figure S1.IGV Snapshot of aligned readsdocx.docx]

**Figure S1.** Snapshot of IGV genome browser showing a portion of the region in scaffold NW_013535362.1. **A**. Reads of the three sequenced populations are identical to the reference genome; **B**. Portion of the region upstream of the total sweep, where only the resistant genotype (R347) maintains identity with the genome while the susceptible lines ALM and FIG have accumulated polymorphisms. The same pattern is see downstream of the total sweep (not shown). Read coverage tracks display gray for same nucleotide and color for nucleotide changes
