# Supplementary material for: Selective Sweeps in a Nutshell: The Genomic Footprint of Rapid Insecticide Resistance Evolution in the Almond Agroecosystem
Source: Genome Biol Evol. 2020 Nov 4;13(1):evaa234. doi: 10.1093/gbe/evaa234 (PMC7850051; doi:10.1093/gbe/evaa234)

**Figure S2.** Alignment of reads for the three sequenced populations to the SPIRL-1966 reference genome in the voltage-gated sodium channel *para* (A), and in the Krüppel-like transcription factor (B), both located in the sweep region in Scaffold NW_013535362.1 of the *Amyelois transitella* genome. Gray color signifies exact base match, colored lines are mutations relative to the reference genome. The arrow in (A) shows the only mutation found in para that correspond to the *kdr* mutation that confers resistance to DDT and pyrethroids.


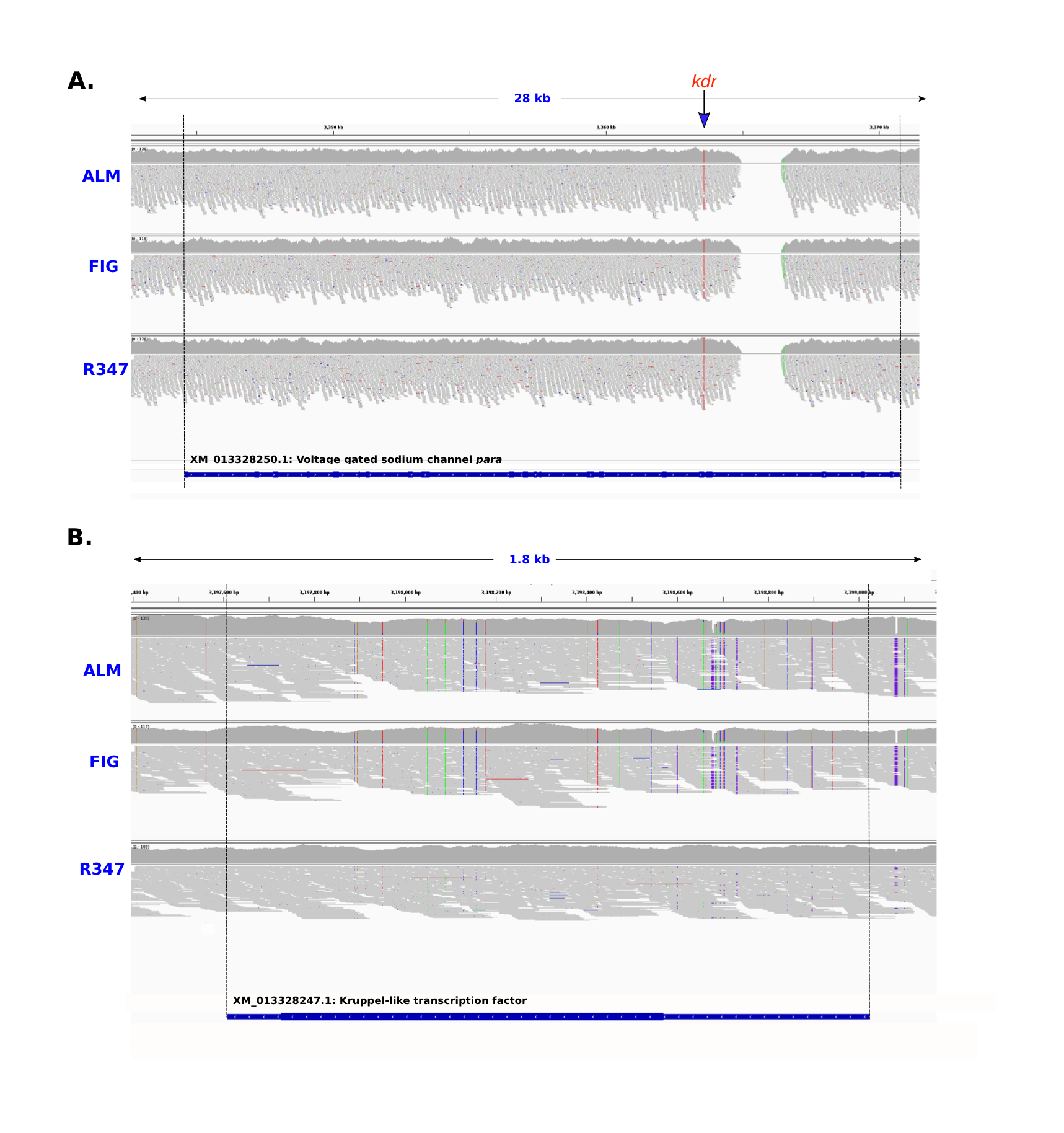

Supplement: evaa234_Supplementary_Data [file evaa234_supplementary_data.zip › Figure S2.Alingments_on_para_and_KL9.docx]
