# Supplementary material for: Selective Sweeps in a Nutshell: The Genomic Footprint of Rapid Insecticide Resistance Evolution in the Almond Agroecosystem
Source: Genome Biol Evol. 2020 Nov 4;13(1):evaa234. doi: 10.1093/gbe/evaa234 (PMC7850051; doi:10.1093/gbe/evaa234)
Supplement: evaa234_Supplementary_Data [file evaa234_supplementary_data.zip › Figure S3. Sanger_sequencing.docx]

**Figure S3.** Sequence of the region flanking the position of the *kdr* mutation in the para gene in ten museum individuals of the SPIRL-1966 strain.
