# Supplementary material for: Selective Sweeps in a Nutshell: The Genomic Footprint of Rapid Insecticide Resistance Evolution in the Almond Agroecosystem
Source: Genome Biol Evol. 2020 Nov 4;13(1):evaa234. doi: 10.1093/gbe/evaa234 (PMC7850051; doi:10.1093/gbe/evaa234)
Supplement: evaa234_Supplementary_Data [file evaa234_supplementary_data.zip › Figure S5.qPCR_results_plots.docx]

**Figure S5.** Quantitative real-time PCR (qPCR) of transcripts encoding cytochrome P450s and the Krüppel-like transcription factor. These genes are contained in the large selective sweep detected in scaffold NW_013535362.1 of the navel orangeworm *A. transitella* populations from the Central Valley in California. The *A. transitella* GADPH gene was used to normalize the CT values. Fold-changes triggered by bifenthrin feeding and due to differences genotypes were calculated relative to the susceptible ALM genotype feeding on control diet (without bifenthrin).
