# Supplementary material for: Selective Sweeps in a Nutshell: The Genomic Footprint of Rapid Insecticide Resistance Evolution in the Almond Agroecosystem
Source: Genome Biol Evol. 2020 Nov 4;13(1):evaa234. doi: 10.1093/gbe/evaa234 (PMC7850051; doi:10.1093/gbe/evaa234)
Supplement: evaa234_Supplementary_Data [file evaa234_supplementary_data.zip › Figure S6.PAML_results_and_tree.docx]

Figure S3. CodeML analyses. The tree shows the gene tree - species tree reconciliation. Duplications that originated the CYP6B tandem in four species of Lepidoptera are circled. Branch nmbers as assigned by CodeML are shown for the branches of interest. Model details and p-values are shown below for the likelihood ratio tests.


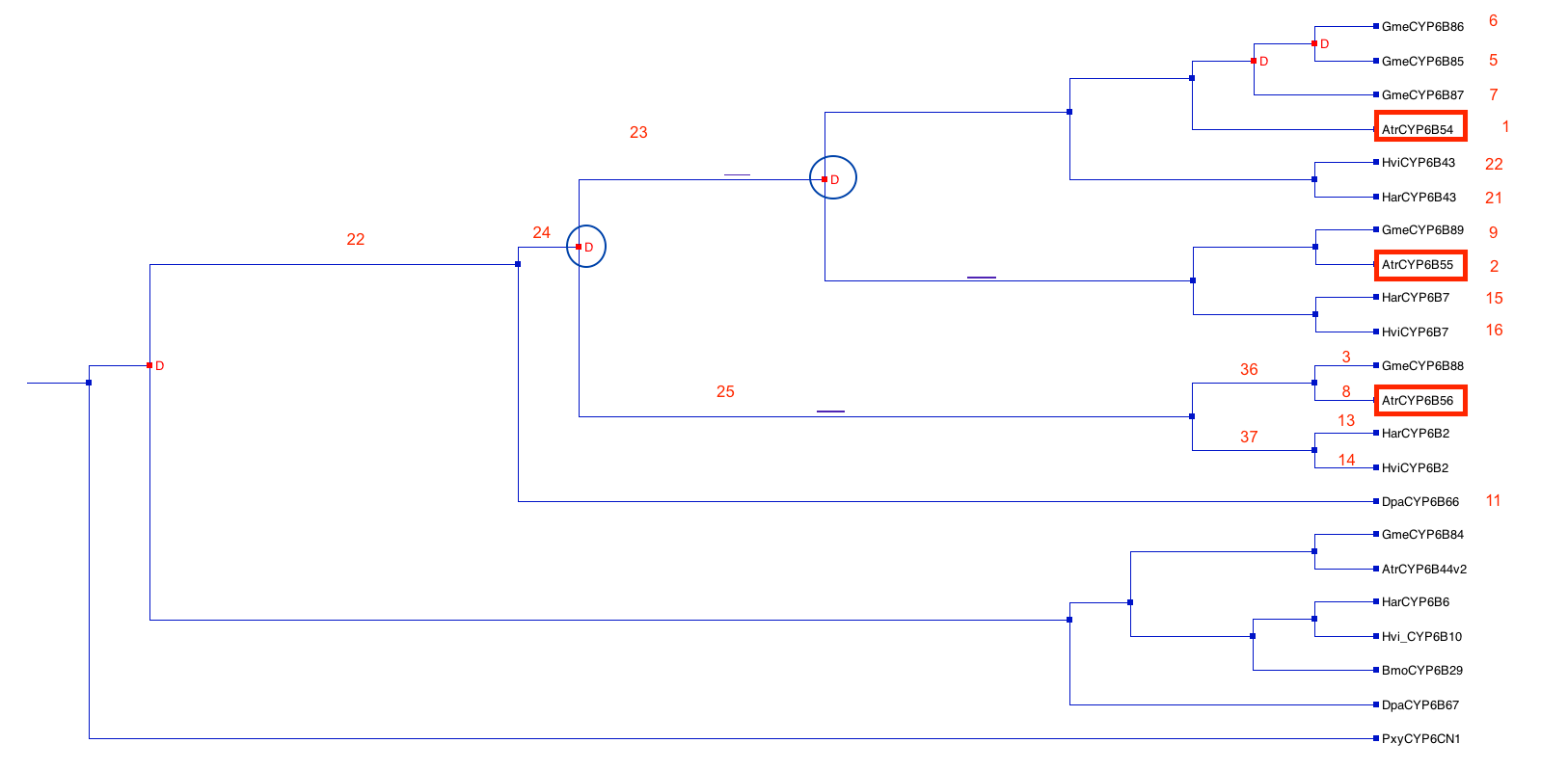


| **Model** | **Branches with different ratio** | **k** | **dN/dS (ω1)** | **dN/dS (ω2)** | **lnL** | **H0 lnL** | **LRT** | **p-value** |  |
| --- | --- | --- | --- | --- | --- | --- | --- | --- | --- |
| 0 | all branches (H0) | 1.6916 | 0.10472 |  | -17155.561 | -17155.561 | 0 | N/A |  |
| 2 | #24 only (burst of positive selection) | 1.6920 | 0.1043 | 0.1145 | -17155.507 | -17155.561 | 0.10781 | 0.74265133 |  |
| 2 | #25 and sub-branches | 1.6931 | 0.10703 | 0.09414 | -17155.087 | -17155.561 | 0.947792 | 0.33028196 |  |
| 2 | #24 branch and all sub-branches | 1.6891 | 0.1224 | 0.0662 | -17144.942 | -17155.561 | 21.237058 | 0.00000406 | ** |
| 2 | #22 only (burst of positive selection) | 1.6867 | 0.10066 | 0.1282 | -17154.481 | -17155.561 | 2.1596 | 0.14168157 |  |
| 2 | #22 and all subbranches | 1.6861 | 0.04989 | 0.13269 | -17132.826 | -17155.561 | 45.46976 | 0.00000000 | ** |
| 2 | #23 and all subranches and tips | 1.6944 | 0.13692 | 0.07699 | -17141.507 | -17155.561 | 28.10716 | 0.00000011 | ** |
