# Supplementary material for: Selective Sweeps in a Nutshell: The Genomic Footprint of Rapid Insecticide Resistance Evolution in the Almond Agroecosystem
Source: Genome Biol Evol. 2020 Nov 4;13(1):evaa234. doi: 10.1093/gbe/evaa234 (PMC7850051; doi:10.1093/gbe/evaa234)

**Figure** **S4. A.** Bifenthrin use in Kern and Madera County almond orchards versus all other pyrethroids reported in the DPR pesticide use records (cyfluthrin, beta-cyfluthrin, (S)-cypermethrin, deltamethrin, esfenvalerate, fenpropathrin, lambda-cyhalothrin, gamma-cyhalothrin, permethrin) from 2006 to 2017.

**B**. Bifenthrin use since its registration in 2006 versus all other pyrethroids reported in the DPR pesticide use records (cyfluthrin, beta-cyfluthrin, (S)-cypermethrin, deltamethrin, esfenvalerate, fenpropathrin, lambda-cyhalothrin, gamma-cyhalothrin, permethrin) in almond orchards from 2006 – 2017.

**A.**


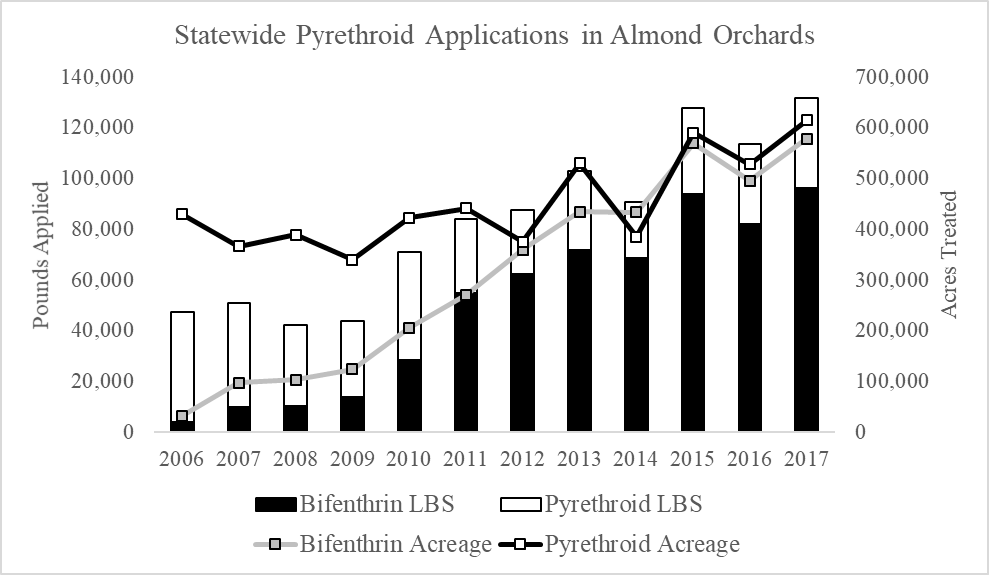


**B.**


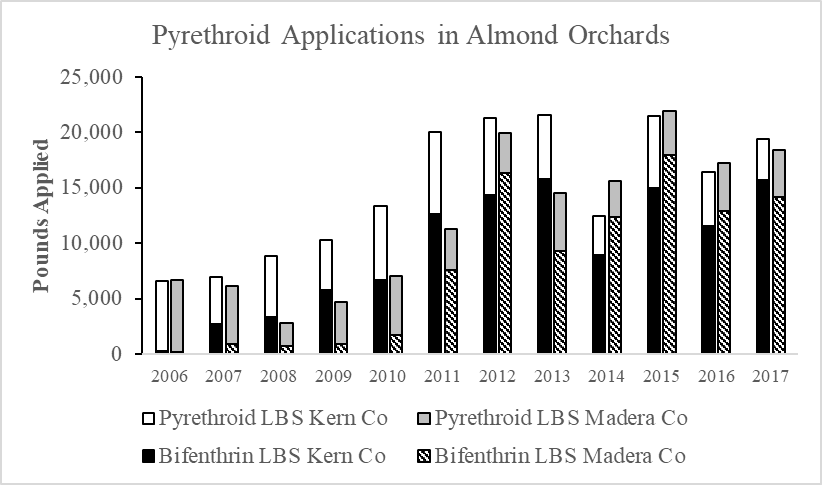

Supplement: evaa234_Supplementary_Data [file evaa234_supplementary_data.zip › Figure S7. Bifenthrin applications historical data.docx]
