# Supplementary material for: Selective Sweeps in a Nutshell: The Genomic Footprint of Rapid Insecticide Resistance Evolution in the Almond Agroecosystem
Source: Genome Biol Evol. 2020 Nov 4;13(1):evaa234. doi: 10.1093/gbe/evaa234 (PMC7850051; doi:10.1093/gbe/evaa234)
Supplement: evaa234_Supplementary_Data [file evaa234_supplementary_data.zip › Supplementary_Methods.docx]

**Supplementary Methods**

**Selective sweeps in a nutshell: the genomic footprints of rapid insecticide resistance evolution in the almond agroecosystem**

**Author and affiliations:**

Bernarda Calla^1^, Mark Demkovich^1^, Joel P. Siegel^3^, João Paulo Gomes Viana^2^, Kim K.O. Walden^1^, Hugh M. Robertson^1^, May R. Berenbaum^1^

**DNA Extraction and sequencing library preparation**

Genomic DNA was extracted from the heads of 100 adult moths (equal sex ratios) Insects were ground in liquid nitrogen, lysed overnight with SDS and Proteinase K, treated with RNase A, and centrifuged in a high-salt solution to precipitate proteins. The DNA was precipitated with ethanol, re-suspended in 10 mM Tris pH 8, and evaluated quantitatively and qualitatively with a Qubit fluorometer (Thermo Fisher Scientific, USA) and checked for degradation on an agarose gel. Subsequently, 2.5 µg of male head DNA were combined with 2.5 µg of female head DNA into a single tube for each of the three strains. Shotgun genomic libraries were prepared with the Hyper Library construction kit (Kapa Biosystems, Wilmington, MA) from equimolar-pooled DNA samples from each of the three populations. Library construction and sequencing were carried out at the W.M. Keck Center of the Roy J. Carver Biotechnology Center at the University of Illinois at Urbana-Champaign.

**RNA-seq**

Differential ethe adapter sequences were trimmed and the 150 nt-long resulting reads were filtered for low quality bases using Trimmomatic [1] with settings to remove the heading and trailing sequences with Phred quality scores below 36.

An indexed reference genome was generated with the –runMode genomeGenerate option within STAR (v. 2.6.0c) software [2], and the trimmed reads were mapped to the assembled reference genome with the paired-end mode in STAR software. Transcript quantification was carried out RSEM v.1.3.0 [3]. After RSEM quantification, gene expression matrices were generated using the “abundance_estimates_to_matrix.pl” from Trinity software (v.2.6.6)[4]. The counts per million (CPM) matrix was used for statistical calculation of differential expression using EdgeR (v.3.4.4) [5]. Cross-sample normalization of read counts was done with the TMM method (trimmed means of M-values) [5, 6], and the normalized counts were used for constructing the heatmap.

**dN/dS with PAML:**

The 22 CYP6Bs nucleotide sequences from seven Lepidoptera species were codon aligned with MUSCLE ([7] as implemented in Geneious v. 9.1.8 [8]. The alignment was trimmed to remove misaligned ends and cleaned to remove unreliable positions. A total of 338 codons were used to calculate a maximum likelihood tree was using RAML with a GTR-Gamma model and 200 bootstrap replications [9]. The tree showed an initial duplication event that separated the CYP6Bs tandem from the CYP6B that is found elsewhere in the genomes of these species.

This gene tree was used with the species tree to infer events of duplication and loss with Notung [10], and the most parsimonious tree topology, together with the codon alignment were further analyzed with the CodeML program from the PAML v.4.8 [11, 12]. Branch models were used, including the neutral (M0) model of one omega value for all branches. The test with M2 model of different omega for different branches (along branches of interest) were compared with M0 and the likelihood ratio test was used to determine the best models compared to M0. The test was corrected for multiple testing with Bonferroni correction.

***Pesticide application data:***

Records of pyrethroid use were accessed through the California Department of Pesticide Regulation (CDPR) - pesticide use annual reports from 1990-2016. Total bifenthrin use in almond orchards was analyzed in Kern County, Madera County, and statewide based on number of applications, pounds of active ingredient, and acres treated from 2006-2016. We also examined records of all pyrethroids applied in almonds from 2000-2016 and compared bifenthrin use relative to all registered pyrethroids by pounds of active ingredient and acres treated.

References:

[1] Bolger, A.M., Usadel, B. & Lohse, M. 2014 Trimmomatic: a flexible trimmer for Illumina sequence data. *Bioinformatics* **30**, 2114-2120. (doi:10.1093/bioinformatics/btu170).

[2] Dobin, A., Davis, C.A., Zaleski, C., Schlesinger, F., Drenkow, J., Chaisson, M., Batut, P., Jha, S. & Gingeras, T.R. 2012 STAR: ultrafast universal RNA-seq aligner. *Bioinformatics* **29**, 15-21. (doi:10.1093/bioinformatics/bts635).

[3] Li, B. & Dewey, C.N. 2011 Rsem: Accurate transcript quantification from rna-seq data with or without a reference genome. *BMC Bioinformatics* **12**, 323. (doi:10.1186/1471-2105-12-323).

[4] Haas, B.J., Papanicolaou, A., Yassour, M., Grabherr, M., Blood, P.D., Bowden, J., Couger, M.B., Eccles, D., Li, B., Lieber, M., et al. 2013 De novo transcript sequence reconstruction from rna-seq using the trinity platform for reference generation and analysis. *Nat. Protocols* **8**, 1494-1512. (doi:10.1038/nprot.2013.084

http://www.nature.com/nprot/journal/v8/n8/abs/nprot.2013.084.html - supplementary-information).

[5] Robinson, M.D., McCarthy, D.J. & Smyth, G.K. 2010 EdgeR: A bioconductor package for differential expression analysis of digital gene expression data. *Bioinformatics* **26**. (doi:10.1093/bioinformatics/btp616).

[6] Robinson, M.D. & Oshlack, A. 2010 A scaling normalization method for differential expression analysis of RNA-seq data. *Genome Biology* **11**, R25. (doi:10.1186/gb-2010-11-3-r25).

[7] Edgar, R.C. 2004 Muscle: Multiple sequence alignment with high accuracy and high throughput. *Nucleic Acids Res.* **32**, 1792-1797. (doi:10.1093/nar/gkh340).

[8] Kearse, M., Moir, R., Wilson, A., Stones-Havas, S., Cheung, M., Sturrock, S., Buxton, S., Cooper, A., Markowitz, S., Duran, C., et al. 2012 Geneious basic: An integrated and extendable desktop software platform for the organization and analysis of sequence data. *Bioinformatics* **28**, 1647-1649. (doi:10.1093/bioinformatics/bts199).

[9] Stamatakis, A. 2014 RaxML version 8: A tool for phylogenetic analysis and post-analysis of large phylogenies. *Bioinformatics* **30**, 1312-1313. (doi:10.1093/bioinformatics/btu033).

[10] Chen, K., Durand, D. & Farach-Colton, M. 2000 Notung: A program for dating gene duplications and optimizing gene family trees. *J. Comput. Biol.* **7**, 429-447. (doi:10.1089/106652700750050871).

[11] Yang, Z. 1996 Maximum-likelihood models for combined analyses of multiple sequence data. *J. Mol. Evol.* **42**, 587-596. (doi:10.1007/bf02352289).

[12] Yang, Z. 2007 PAML 4: Phylogenetic analysis by maximum likelihood. *Mol. Biol. Evol.* **24**, 1586-1591. (doi:10.1093/molbev/msm088).
