# Supplementary material for: Selective Sweeps in a Nutshell: The Genomic Footprint of Rapid Insecticide Resistance Evolution in the Almond Agroecosystem
Source: Genome Biol Evol. 2020 Nov 4;13(1):evaa234. doi: 10.1093/gbe/evaa234 (PMC7850051; doi:10.1093/gbe/evaa234)
Supplement: evaa234_Supplementary_Data [file evaa234_supplementary_data.zip › Table S1.docx]

**Table S1.** Pooled DNA re-sequencing results.

| **Strain** | **Library Name** | **Number of reads** | **Read length** | **Paired** | **Haploid genome length** | **Coverage** |
| --- | --- | --- | --- | --- | --- | --- |
| **Almond** | Almond_AAGGCCGTCA_L006_R1_001.fastq | 120,568,394 | 150 | 2 | 406,468,287 | 88.987 |
| **Fig** | Fig_AAGATCTGAG_L006_R1_001.fastq | 112,371,554 | 150 | 2 | 406,468,287 | 82.938 |
| **R347** | R347_AAGGTGCCTG_L006_R1_001.fastq | 119,586,826 | 150 | 2 | 406,468,287 | 88.263 |
|  | **Total Reads (paired)** | **705,053,548** |  |  |  | **260.1876594** |
