# Supplementary material for: Selective Sweeps in a Nutshell: The Genomic Footprint of Rapid Insecticide Resistance Evolution in the Almond Agroecosystem
Source: Genome Biol Evol. 2020 Nov 4;13(1):evaa234. doi: 10.1093/gbe/evaa234 (PMC7850051; doi:10.1093/gbe/evaa234)
Supplement: evaa234_Supplementary_Data [file evaa234_supplementary_data.zip › Table S2.docx]

| **Table S2. Top and bottom of Tajima's D values for each population** | | | | |
| --- | --- | --- | --- | --- |
|  | **bottom 1% range** | | **top 1% range** | |
| **ALM** | -2.46122 | -1.25244 | 0.9528931 | 2.2623047 |
| **FIG** | -2.428976 | 1.283733 | 0.9362361 | 2.2729293 |
| **R347** | -2.501666 | -1.277181 | 1.237428 | 2.587189 |
