# Supplementary material for: Selective Sweeps in a Nutshell: The Genomic Footprint of Rapid Insecticide Resistance Evolution in the Almond Agroecosystem
Source: Genome Biol Evol. 2020 Nov 4;13(1):evaa234. doi: 10.1093/gbe/evaa234 (PMC7850051; doi:10.1093/gbe/evaa234)
Supplement: evaa234_Supplementary_Data [file evaa234_supplementary_data.zip › Table S3.docx]

**Table S3.** Nucleotide diversity for each gene across scaffold NW_013535362.1. The number of SNPs, read coverage and the calculated Tajima’s π are shown for each of the sequenced populations.

| **ID** | **gene_ID** | **Star** | **End** | **gene_name** | **ALM number of SNPS** | **ALM coverage** | **ALM_pi** | **FIG number of SNPS** | **FIG coverage** | **Fig_pi** | **R347 number of SNPS** | **R347 coverage** | **R347_pi** |
| --- | --- | --- | --- | --- | --- | --- | --- | --- | --- | --- | --- | --- | --- |
| rna4429 | XM_013328369.1 | 3399243 | 3401314 | CYPB56 | 2 | 0.966 | 0.000119055 | 3 | 0.932 | 0.000146796 | 0 | 0.999 | 0 |
| rna4431 | XM_013328342.1 | 3413197 | 3420607 | protein ariadne-1%2C transcript variant X2 | 11 | 0.929 | 0.000169131 | 19 | 0.927 | 0.000340522 | 1 | 0.891 | 0.000012382 |
| rna4443 | XM_013328236.1 | 3549071 | 3550177 | protein kish-A | 0 | 0.536 | 0 | 5 | 0.921 | 0.00047616 | 1 | 1 | 0.000075361 |
| rna4430 | XM_013328341.1 | 3403909 | 3420607 | protein ariadne-1%2C transcript variant X1 | 19 | 0.851 | 0.000133218 | 43 | 0.92 | 0.000287535 | 13 | 0.865 | 0.000078983 |
| rna4427 | XM_013328250.1 | 3344559 | 3350148 | sodium channel protein para%2C transcript variant X2 | 1 | 0.873 | 0.000016742 | 7 | 0.958 | 0.000106802 | 6 | 0.838 | 0.000121451 |
| rna4426 | XM_013328249.1 | 3344559 | 3370779 | sodium channel protein para%2C transcript variant X1 | 33 | 0.829 | 0.000155447 | 41 | 0.898 | 0.000163303 | 30 | 0.826 | 0.000129155 |
| rna4444 | XM_013328238.1 | 3550487 | 3554188 | protein prenyltransferase alpha subunit repeat-containing protein 1 | 21 | 0.797 | 0.000809689 | 33 | 0.842 | 0.001342665 | 5 | 0.763 | 0.000158307 |
| rna4432 | XM_013328377.1 | 3421473 | 3423241 | uncharacterized LOC106129721 | 3 | 0.939 | 0.000147596 | 4 | 0.993 | 0.000186148 | 3 | 0.949 | 0.000168929 |
| rna4447 | XM_013328187.1 | 3745239 | 3796518 | protein unc-13 homolog A | 623 | 0.685 | 0.002149737 | 728 | 0.736 | 0.002379027 | 75 | 0.764 | 0.000174787 |
| rna4448 | XM_013328228.1 | 3800374 | 3817393 | actin-binding Rho-activating protein-like | 214 | 0.665 | 0.002477321 | 304 | 0.707 | 0.003822464 | 27 | 0.729 | 0.000196256 |
| rna4433 | XM_013328378.1 | 3423369 | 3430390 | isoleucine--tRNA ligase%2C cytoplasmic | 11 | 0.683 | 0.000210947 | 32 | 0.746 | 0.000671049 | 15 | 0.719 | 0.000265044 |
| rna4445 | XM_013328171.1 | 3584697 | 3630468 | uncharacterized LOC106129575 | 485 | 0.659 | 0.001909073 | 652 | 0.653 | 0.002842798 | 97 | 0.675 | 0.000319536 |
| rna4434 | XM_013328269.1 | 3433176 | 3489393 | dmX-like protein 2 | 408 | 0.637 | 0.001241575 | 449 | 0.667 | 0.001385853 | 121 | 0.646 | 0.000323764 |
| rna4442 | XM_013328235.1 | 3529722 | 3548710 | ras GTPase-activating protein 1 | 173 | 0.693 | 0.001636741 | 241 | 0.761 | 0.00197034 | 52 | 0.741 | 0.00035081 |
| rna4428 | XM_013328169.1 | 3387986 | 3396394 | CYPB54-55 | 31 | 0.877 | 0.000374048 | 67 | 0.945 | 0.000832382 | 30 | 0.889 | 0.000397261 |
| rna4449 | XM_013328172.1 | 3826528 | 3849030 | gonadotropin-releasing hormone II receptor-like | 326 | 0.757 | 0.002598439 | 460 | 0.75 | 0.004254889 | 87 | 0.778 | 0.00047073 |
| rna4438 | XM_013328231.1 | 3490744 | 3524106 | endophilin-A%2C transcript variant X1 | 288 | 0.792 | 0.001364922 | 273 | 0.854 | 0.001120561 | 106 | 0.847 | 0.000511522 |
| rna4440 | XM_013328232.1 | 3490744 | 3524106 | endophilin-A%2C transcript variant X2 | 288 | 0.792 | 0.001364922 | 273 | 0.854 | 0.001120561 | 106 | 0.847 | 0.000511522 |
| rna4439 | XM_013328233.1 | 3490744 | 3524106 | endophilin-A%2C transcript variant X3 | 288 | 0.792 | 0.001364922 | 273 | 0.854 | 0.001120561 | 106 | 0.847 | 0.000511522 |
| rna4446 | XM_013328188.1 | 3631722 | 3638019 | uncharacterized LOC106129590 | 57 | 0.629 | 0.001609691 | 75 | 0.677 | 0.002214825 | 24 | 0.611 | 0.000589032 |
| rna4455 | XM_013328217.1 | 4270710 | 4272681 | tyrosine--tRNA ligase%2C cytoplasmic | 15 | 0.589 | 0.002191836 | 22 | 0.915 | 0.002546646 | 8 | 0.902 | 0.000683768 |
| rna4390 | XM_013328351.1 | 2580309 | 2581660 | UDP-GlcNAc:betaGal beta-1%2C3-N-acetylglucosaminyltransferase-like protein 1 | 7 | 0.949 | 0.001699828 | 5 | 0.919 | 0.00140442 | 6 | 0.865 | 0.001191301 |
| rna4418 | XM_013328423.1 | 3159383 | 3164900 | uncharacterized LOC106129757 | 26 | 0.673 | 0.001504173 | 21 | 0.711 | 0.001399366 | 25 | 0.73 | 0.001236382 |
| rna4337 | XM_013328277.1 | 1505319 | 1507681 | conserved oligomeric Golgi complex subunit 5 | 24 | 0.647 | 0.003325352 | 36 | 0.924 | 0.004487335 | 12 | 0.89 | 0.001648673 |
| rna4425 | XM_013328229.1 | 3304902 | 3307354 | phosphomevalonate kinase | 28 | 0.914 | 0.002209508 | 28 | 0.967 | 0.002526449 | 17 | 0.712 | 0.001704432 |
| rna4413 | XM_013328280.1 | 3097871 | 3099727 | GPI mannosyltransferase 2%2C transcript variant X2 | 10 | 0.893 | 0.001245343 | 7 | 0.907 | 0.000848715 | 6 | 0.912 | 0.001815043 |
| rna4414 | XM_013328279.1 | 3097905 | 3099727 | GPI mannosyltransferase 2%2C transcript variant X1 | 9 | 0.891 | 0.001221095 | 7 | 0.906 | 0.000866193 | 6 | 0.919 | 0.001834536 |
| rna4441 | XM_013328234.1 | 3525167 | 3528547 | polyubiquitin-C | 51 | 0.639 | 0.002994905 | 40 | 0.676 | 0.002750336 | 26 | 0.67 | 0.002126739 |
| rna4391 | XM_013328352.1 | 2581733 | 2585900 | stress-induced-phosphoprotein 1-like | 50 | 0.893 | 0.00302288 | 54 | 0.942 | 0.003067504 | 31 | 0.899 | 0.002209818 |
| rna4422 | XM_013328326.1 | 3204704 | 3279423 | protein groucho%2C transcript variant X1 | 512 | 0.615 | 0.00255649 | 570 | 0.629 | 0.002639457 | 492 | 0.618 | 0.00259059 |
| rna4451 | XM_013328392.1 | 4090436 | 4108229 | uncharacterized LOC106129735 | 333 | 0.696 | 0.007056351 | 396 | 0.76 | 0.006815791 | 213 | 0.745 | 0.002636165 |
| rna4423 | XM_013328328.1 | 3204704 | 3258508 | protein groucho%2C transcript variant X3 | 410 | 0.604 | 0.002993113 | 461 | 0.614 | 0.003024779 | 386 | 0.605 | 0.002909226 |
| rna4424 | XM_013328327.1 | 3204704 | 3256842 | protein groucho%2C transcript variant X2 | 393 | 0.593 | 0.003028286 | 443 | 0.603 | 0.003050611 | 369 | 0.593 | 0.002923422 |
| rna4383 | XM_013328409.1 | 2419495 | 2425584 | putative elongator complex protein 1%2C transcript variant X1 | 90 | 0.895 | 0.004315263 | 94 | 0.923 | 0.004142576 | 66 | 0.893 | 0.003205471 |
| rna4384 | XM_013328410.1 | 2419495 | 2425584 | putative elongator complex protein 1%2C transcript variant X2 | 90 | 0.895 | 0.004315263 | 94 | 0.923 | 0.004142576 | 66 | 0.893 | 0.003205471 |
| rna4421 | XM_013328247.1 | 3197609 | 3199024 | Krueppel-like factor 9 | 24 | 0.833 | 0.005024417 | 23 | 0.898 | 0.004206684 | 23 | 0.965 | 0.003498993 |
| rna4450 | XM_013328189.1 | 3852565 | 4034421 | small conductance calcium-activated potassium channel protein | 4722 | 0.628 | 0.008780796 | 5092 | 0.629 | 0.00959187 | 2798 | 0.693 | 0.003576904 |
| rna4408 | XM_013328167.1 | 3044389 | 3048122 | uncharacterized LOC106129572 | 45 | 0.801 | 0.002517566 | 27 | 0.887 | 0.001446331 | 36 | 0.948 | 0.003757452 |
| rna4453 | XM_013328173.1 | 4247890 | 4251407 | uncharacterized protein DDB_G0286591-like | 71 | 0.792 | 0.005590469 | 75 | 0.86 | 0.005145974 | 42 | 0.849 | 0.003790941 |
| rna4530 | XM_013328414.1 | 5881767 | 5883523 | DTW domain-containing protein 2 | 25 | 0.789 | 0.004663524 | 25 | 0.873 | 0.004628095 | 20 | 0.701 | 0.004146948 |
| rna4508 | XM_013328386.1 | 5483318 | 5487648 | odorant receptor 4-like | 138 | 0.779 | 0.00653974 | 120 | 0.773 | 0.006490545 | 83 | 0.822 | 0.004175595 |
| rna4331 | XM_013328225.1 | 1473705 | 1476159 | m7GpppN-mRNA hydrolase | 29 | 0.927 | 0.003160619 | 27 | 0.933 | 0.003546826 | 21 | 0.901 | 0.004217887 |
| rna4501 | XM_013328240.1 | 5313693 | 5315051 | mitochondrial inner membrane protein OXA1L | 25 | 0.993 | 0.005148463 | 17 | 0.722 | 0.004908737 | 15 | 0.712 | 0.004220994 |
| rna4517 | XM_013328185.1 | 5724518 | 5727850 | uncharacterized LOC106129587 | 34 | 0.743 | 0.00451717 | 54 | 0.939 | 0.004745916 | 41 | 0.852 | 0.004393735 |
| rna4405 | XM_013328243.1 | 3002489 | 3003978 | cytochrome c oxidase assembly protein COX16 homolog%2C mitochondrial | 27 | 0.712 | 0.006641446 | 19 | 0.83 | 0.005655548 | 23 | 0.85 | 0.004474807 |
| rna4456 | XM_013328325.1 | 4275218 | 4323747 | RNA polymerase II elongation factor ELL | 966 | 0.783 | 0.006161608 | 978 | 0.829 | 0.005349858 | 571 | 0.798 | 0.004598154 |
| rna4396 | XM_013328380.1 | 2593141 | 2603646 | myelin expression factor 2-like%2C transcript variant X2 | 145 | 0.608 | 0.007917822 | 152 | 0.588 | 0.008019857 | 110 | 0.601 | 0.004679833 |
| rna4321 | XM_013328346.1 | 1399170 | 1408734 | uncharacterized LOC106129698 | 161 | 0.71 | 0.006730182 | 160 | 0.689 | 0.006518281 | 85 | 0.622 | 0.004759801 |
| rna4330 | XM_013328241.1 | 1471195 | 1473165 | syntaxin-18 | 30 | 0.758 | 0.007262027 | 33 | 0.697 | 0.008038343 | 18 | 0.688 | 0.004929163 |
| rna4420 | XM_013328361.1 | 3164948 | 3193977 | unconventional myosin-IXa-like%2C transcript variant X1 | 579 | 0.756 | 0.005938819 | 548 | 0.797 | 0.005037003 | 496 | 0.774 | 0.005021211 |
| rna4419 | XM_013328362.1 | 3164948 | 3193977 | unconventional myosin-IXa-like%2C transcript variant X2 | 579 | 0.756 | 0.005938819 | 548 | 0.797 | 0.005037003 | 496 | 0.774 | 0.005021211 |
| rna4338 | XM_013328278.1 | 1508705 | 1518258 | GTPase HRas | 131 | 0.659 | 0.006153678 | 126 | 0.693 | 0.006000635 | 100 | 0.672 | 0.005151254 |
| rna4340 | XM_013328422.1 | 1519937 | 1520766 | dolichol-phosphate mannosyltransferase subunit 3%2C transcript variant X2 | 17 | 0.882 | 0.007364632 | 18 | 0.861 | 0.007774272 | 12 | 0.972 | 0.005275164 |
| rna4454 | XM_013328174.1 | 4268658 | 4270209 | uncharacterized LOC106129578 | 43 | 0.922 | 0.007847321 | 45 | 0.918 | 0.0065733 | 41 | 0.852 | 0.005321223 |
| rna4536 | XM_013328244.1 | 6012409 | 6013623 | uncharacterized protein C19orf52 | 20 | 0.737 | 0.004995216 | 31 | 0.891 | 0.007180479 | 19 | 0.792 | 0.005331318 |
| rna4521 | XM_013328393.1 | 5745189 | 5751175 | gbkey=mRNA | 85 | 0.62 | 0.005618301 | 85 | 0.623 | 0.005251222 | 94 | 0.693 | 0.005670913 |
| rna4393 | XM_013328382.1 | 2593138 | 2598806 | myelin expression factor 2-like%2C transcript variant X4 | 79 | 0.626 | 0.008517212 | 81 | 0.592 | 0.008954112 | 65 | 0.617 | 0.005697184 |
| rna4298 | XM_013328239.1 | 880578 | 883070 | protein bud22 | 49 | 0.868 | 0.005870367 | 49 | 0.893 | 0.005227974 | 44 | 0.878 | 0.005884998 |
| rna4339 | XM_013328421.1 | 1519661 | 1520744 | dolichol-phosphate mannosyltransferase subunit 3%2C transcript variant X1 | 24 | 0.885 | 0.008233695 | 25 | 0.869 | 0.008556691 | 16 | 0.956 | 0.006077829 |
| rna4378 | XM_013328165.1 | 2399919 | 2400599 | uncharacterized LOC106129569 | 13 | 0.921 | 0.007158843 | 15 | 0.938 | 0.007078859 | 11 | 0.927 | 0.006090894 |
| rna4496 | XM_013328333.1 | 5271861 | 5273537 | neugrin | 32 | 0.769 | 0.0075507 | 40 | 0.908 | 0.007070333 | 30 | 0.882 | 0.006332797 |
| rna4380 | XM_013328417.1 | 2410837 | 2412344 | beta-ureidopropionase-like | 25 | 0.854 | 0.005200978 | 29 | 0.936 | 0.005376323 | 23 | 0.885 | 0.006505778 |
| rna4381 | XM_013328418.1 | 2412449 | 2413979 | eukaryotic translation initiation factor 2 subunit 2 | 19 | 0.673 | 0.004709099 | 25 | 0.943 | 0.004881273 | 23 | 0.94 | 0.006689415 |
| rna4406 | XM_013328367.1 | 3012239 | 3045757 | tyrosine-protein kinase CSK%2C transcript variant X1 | 558 | 0.648 | 0.007588042 | 530 | 0.702 | 0.006464824 | 463 | 0.68 | 0.006805918 |
| rna4385 | XM_013328242.1 | 2425702 | 2429091 | leucine-rich repeat protein 1 | 68 | 0.792 | 0.007598856 | 67 | 0.804 | 0.006949772 | 53 | 0.814 | 0.006885488 |
| rna4392 | XM_013328399.1 | 2587577 | 2592737 | integrator complex subunit 3 homolog | 84 | 0.716 | 0.008476183 | 90 | 0.864 | 0.007165859 | 88 | 0.719 | 0.007058129 |
| rna4511 | XM_013328182.1 | 5504764 | 5505982 | lipase 1-like | 17 | 0.587 | 0.007667123 | 31 | 0.974 | 0.009696404 | 27 | 0.996 | 0.007102324 |
| rna4359 | XM_013328358.1 | 1827009 | 1830838 | vacuolar protein sorting-associated protein 33B%2C transcript variant X2 | 73 | 0.552 | 0.01019639 | 60 | 0.46 | 0.011606181 | 48 | 0.539 | 0.007282337 |
| rna4360 | XM_013328357.1 | 1827979 | 1830838 | vacuolar protein sorting-associated protein 33B%2C transcript variant X1 | 73 | 0.74 | 0.01019639 | 60 | 0.616 | 0.011606181 | 48 | 0.722 | 0.007282337 |
| rna4300 | XM_013328258.1 | 887424 | 889772 | ribose-phosphate pyrophosphokinase 2 | 42 | 0.755 | 0.007613161 | 43 | 0.858 | 0.006280155 | 43 | 0.883 | 0.007461008 |
| rna4348 | XM_013328291.1 | 1577783 | 1581428 | BTB/POZ domain-containing protein 2-like%2C transcript variant X1 | 81 | 0.68 | 0.011976623 | 81 | 0.681 | 0.011489997 | 64 | 0.672 | 0.00752791 |
| rna4407 | XM_013328368.1 | 3019509 | 3045757 | tyrosine-protein kinase CSK%2C transcript variant X2 | 492 | 0.681 | 0.008041054 | 472 | 0.742 | 0.006853115 | 415 | 0.723 | 0.007566126 |
| rna4452 | XM_013328198.1 | 4240437 | 4246388 | extensin-like | 260 | 0.789 | 0.015132831 | 260 | 0.809 | 0.013739108 | 178 | 0.84 | 0.007591015 |
| rna4389 | XM_013328350.1 | 2572396 | 2580080 | gbkey=mRNA | 148 | 0.667 | 0.00859795 | 151 | 0.665 | 0.008366142 | 130 | 0.68 | 0.007648406 |
| rna4354 | XM_013328201.1 | 1651101 | 1653941 | alpha-(1%2C3)-fucosyltransferase C-like | 98 | 0.778 | 0.012263638 | 106 | 0.841 | 0.013055453 | 63 | 0.659 | 0.007756923 |
| rna4534 | XM_013328246.1 | 6000856 | 6002196 | uncharacterized LOC106129643 | 27 | 0.961 | 0.00609803 | 26 | 0.936 | 0.005062659 | 27 | 0.919 | 0.00785931 |
| rna4493 | XM_013328336.1 | 5236222 | 5238866 | uncharacterized LOC106129692 | 67 | 0.783 | 0.008596993 | 71 | 0.952 | 0.008128525 | 65 | 0.846 | 0.007874551 |
| rna4525 | XM_013328271.1 | 5775698 | 5788089 | uncharacterized LOC106129662%2C transcript variant X2 | 196 | 0.592 | 0.007924001 | 204 | 0.625 | 0.007401777 | 207 | 0.574 | 0.0080822 |
| rna4526 | XM_013328270.1 | 5775698 | 5786474 | uncharacterized LOC106129662%2C transcript variant X1 | 196 | 0.681 | 0.007924001 | 204 | 0.719 | 0.007403688 | 207 | 0.659 | 0.0080822 |
| rna4500 | XM_013328224.1 | 5310573 | 5313063 | U3 small nucleolar RNA-associated protein 15 homolog | 52 | 0.679 | 0.009316192 | 75 | 0.933 | 0.008723168 | 45 | 0.729 | 0.00821631 |
| rna4522 | XM_013328200.1 | 5753445 | 5759187 | uncharacterized LOC106129599 | 106 | 0.669 | 0.008081293 | 79 | 0.596 | 0.006874992 | 82 | 0.542 | 0.008634491 |
| rna4281 | XM_013328149.1 | 70277 | 73054 | uncharacterized LOC106129551 | 70 | 0.949 | 0.006904584 | 76 | 0.979 | 0.00770778 | 78 | 0.967 | 0.00865995 |
| rna4347 | XM_013328292.1 | 1575910 | 1581428 | BTB/POZ domain-containing protein 2-like%2C transcript variant X2 | 116 | 0.576 | 0.012664871 | 119 | 0.583 | 0.012238872 | 96 | 0.565 | 0.009015599 |
| rna4507 | XM_013328387.1 | 5479669 | 5483229 | pancreatic lipase-related protein 2-like | 112 | 0.858 | 0.010547971 | 129 | 0.906 | 0.011299822 | 95 | 0.929 | 0.009177413 |
| rna4386 | XM_013328214.1 | 2429317 | 2434186 | G kinase-anchoring protein 1-like | 132 | 0.752 | 0.012244815 | 128 | 0.73 | 0.011291087 | 95 | 0.724 | 0.009191826 |
| rna4328 | XM_013328196.1 | 1466142 | 1467679 | RNA-binding protein NOB1 | 35 | 0.888 | 0.009440154 | 36 | 0.916 | 0.009234366 | 30 | 0.845 | 0.009248617 |
| rna4343 | XM_013328379.1 | 1553769 | 1557284 | membrane-bound alkaline phosphatase-like | 74 | 0.617 | 0.008592208 | 76 | 0.608 | 0.009571366 | 57 | 0.588 | 0.009338058 |
| rna4403 | XM_013328219.1 | 2977372 | 2991119 | ADAM 17-like protease | 242 | 0.457 | 0.011319361 | 219 | 0.5 | 0.008955323 | 195 | 0.512 | 0.009365391 |
| rna4345 | XM_013328290.1 | 1563041 | 1565652 | membrane-bound alkaline phosphatase-like | 66 | 0.873 | 0.009084757 | 62 | 0.892 | 0.007516861 | 55 | 0.755 | 0.010169592 |
| rna4409 | XM_013328206.1 | 3049409 | 3053897 | DET1 homolog | 92 | 0.804 | 0.0053506 | 79 | 0.875 | 0.003573386 | 82 | 0.766 | 0.010415605 |
| rna4382 | XM_013328215.1 | 2415442 | 2418993 | 28S ribosomal protein S30%2C mitochondrial | 129 | 0.852 | 0.013025224 | 125 | 0.84 | 0.012615374 | 99 | 0.787 | 0.010437139 |
| rna4531 | XM_013328415.1 | 5884495 | 5886643 | 40S ribosomal protein S15 | 66 | 0.752 | 0.011841779 | 74 | 0.872 | 0.010363965 | 61 | 0.874 | 0.010457711 |
| rna4387 | XM_013328412.1 | 2436696 | 2532139 | A disintegrin and metalloproteinase with thrombospondin motifs 7-like | 2049 | 0.597 | 0.011803109 | 2047 | 0.617 | 0.011169004 | 1781 | 0.604 | 0.010484045 |
| rna4529 | XM_013328186.1 | 5830408 | 5878979 | uncharacterized LOC106129588 | 1115 | 0.587 | 0.010991996 | 1164 | 0.586 | 0.011132651 | 1045 | 0.581 | 0.011062135 |
| rna4467 | XM_013328376.1 | 4842481 | 4850978 | dual specificity mitogen-activated protein kinase kinase dSOR1 | 286 | 0.812 | 0.01048265 | 292 | 0.873 | 0.009530437 | 234 | 0.817 | 0.011341106 |
| rna4352 | XM_013328364.1 | 1632464 | 1635170 | putative nuclease HARBI1 | 75 | 0.743 | 0.01204676 | 91 | 0.762 | 0.012629692 | 65 | 0.685 | 0.011566315 |
| rna4400 | XM_013328253.1 | 2883596 | 2887227 | uncharacterized LOC106129648 | 135 | 0.766 | 0.015635148 | 137 | 0.811 | 0.01543218 | 102 | 0.792 | 0.011600085 |
| rna4329 | XM_013328197.1 | 1467658 | 1470732 | UPF0528 protein CG10038 | 93 | 0.84 | 0.014087576 | 109 | 0.801 | 0.014739893 | 57 | 0.712 | 0.011617053 |
| rna4461 | XM_013328287.1 | 4473947 | 4534011 | uncharacterized LOC106129672 | 2056 | 0.697 | 0.013389586 | 2151 | 0.729 | 0.012438962 | 1599 | 0.701 | 0.01164602 |
| rna4509 | XM_013328180.1 | 5490242 | 5493926 | odorant receptor 4-like | 163 | 0.842 | 0.015328105 | 149 | 0.796 | 0.014657463 | 135 | 0.875 | 0.011770148 |
| rna4460 | XM_013328288.1 | 4419275 | 4437090 | protein giant-lens | 483 | 0.573 | 0.013003857 | 487 | 0.576 | 0.012881567 | 421 | 0.594 | 0.012005888 |
| rna4416 | XM_013328281.1 | 3101631 | 3107700 | methyl-CpG-binding domain protein 3%2C transcript variant X1 | 141 | 0.739 | 0.006844267 | 119 | 0.823 | 0.005241431 | 121 | 0.717 | 0.012030669 |
| rna4415 | XM_013328282.1 | 3101631 | 3107700 | methyl-CpG-binding domain protein 3%2C transcript variant X2 | 141 | 0.739 | 0.006844267 | 119 | 0.823 | 0.005241431 | 121 | 0.717 | 0.012030669 |
| rna4361 | XM_013328345.1 | 1831183 | 1847961 | probable isocitrate dehydrogenase [NAD] subunit alpha%2C mitochondrial%2C transcript variant X3 | 432 | 0.581 | 0.01368344 | 454 | 0.619 | 0.013274785 | 387 | 0.614 | 0.012033377 |
| rna4411 | XM_013328168.1 | 3067370 | 3078948 | tubulin polyglutamylase TTLL13-like | 262 | 0.693 | 0.006962775 | 211 | 0.671 | 0.00557638 | 260 | 0.689 | 0.012051446 |
| rna4362 | XM_013328343.1 | 1831299 | 1847961 | probable isocitrate dehydrogenase [NAD] subunit alpha%2C mitochondrial%2C transcript variant X1 | 430 | 0.579 | 0.013766682 | 453 | 0.617 | 0.013375931 | 385 | 0.611 | 0.012077437 |
| rna4506 | XM_013328203.1 | 5471655 | 5477166 | pancreatic lipase-related protein 2-like | 123 | 0.432 | 0.016173196 | 137 | 0.444 | 0.017888798 | 111 | 0.46 | 0.012177467 |
| rna4342 | XM_013328158.1 | 1549021 | 1552044 | membrane-bound alkaline phosphatase-like | 95 | 0.765 | 0.011345937 | 99 | 0.908 | 0.009984343 | 97 | 0.943 | 0.012457168 |
| rna4510 | XM_013328181.1 | 5500322 | 5503424 | putative odorant receptor 92a | 191 | 0.872 | 0.023127907 | 184 | 0.869 | 0.021780068 | 144 | 0.864 | 0.012824788 |
| rna4333 | XM_013328427.1 | 1476505 | 1484339 | glycosylated lysosomal membrane protein B-like%2C transcript variant X2 | 200 | 0.704 | 0.010096267 | 221 | 0.727 | 0.011198945 | 181 | 0.648 | 0.012865083 |
| rna4332 | XM_013328426.1 | 1476505 | 1484348 | glycosylated lysosomal membrane protein B-like%2C transcript variant X1 | 201 | 0.704 | 0.010105206 | 221 | 0.727 | 0.011187163 | 182 | 0.648 | 0.012878185 |
| rna4350 | XM_013328300.1 | 1621486 | 1629660 | monocarboxylate transporter 1-like%2C transcript variant X1 | 256 | 0.681 | 0.015368042 | 244 | 0.645 | 0.015738436 | 227 | 0.68 | 0.013095432 |
| rna4537 | XM_013328428.1 | 6014289 | 6025097 | exception=annotated by transcript or proteomic data | 315 | 0.696 | 0.011987582 | 321 | 0.726 | 0.011711206 | 328 | 0.71 | 0.013096788 |
| rna4322 | XM_013328354.1 | 1415367 | 1429459 | zinc finger protein 135-like%2C transcript variant X2 | 453 | 0.594 | 0.018622603 | 476 | 0.59 | 0.018443142 | 267 | 0.517 | 0.013110547 |
| rna4357 | XM_013328161.1 | 1795791 | 1810707 | uncharacterized LOC106129566 | 526 | 0.66 | 0.018768043 | 519 | 0.7 | 0.017989018 | 408 | 0.622 | 0.013126036 |
| rna4476 | XM_013328309.1 | 5164520 | 5213764 | casein kinase I isoform gamma-3%2C transcript variant X6 | 1778 | 0.689 | 0.01438096 | 1813 | 0.728 | 0.014184591 | 1446 | 0.729 | 0.01322272 |
| rna4477 | XM_013328313.1 | 5164520 | 5213764 | casein kinase I isoform gamma-3%2C transcript variant X9 | 1778 | 0.689 | 0.01438096 | 1813 | 0.728 | 0.014184591 | 1446 | 0.729 | 0.01322272 |
| rna4482 | XM_013328304.1 | 5164520 | 5213751 | casein kinase I isoform gamma-3%2C transcript variant X2 | 1778 | 0.689 | 0.014386049 | 1813 | 0.728 | 0.014189739 | 1446 | 0.729 | 0.013227508 |
| rna4481 | XM_013328305.1 | 5164520 | 5213751 | casein kinase I isoform gamma-3%2C transcript variant X3 | 1778 | 0.689 | 0.014386049 | 1813 | 0.728 | 0.014189739 | 1446 | 0.729 | 0.013227508 |
| rna4480 | XM_013328310.1 | 5164520 | 5213751 | casein kinase I isoform gamma-3%2C transcript variant X7 | 1778 | 0.689 | 0.014386049 | 1813 | 0.728 | 0.014189739 | 1446 | 0.729 | 0.013227508 |
| rna4479 | XM_013328311.1 | 5164520 | 5213751 | casein kinase I isoform gamma-3%2C transcript variant X8 | 1778 | 0.689 | 0.014386049 | 1813 | 0.728 | 0.014189739 | 1446 | 0.729 | 0.013227508 |
| rna4478 | XM_013328314.1 | 5164520 | 5213751 | casein kinase I isoform gamma-3%2C transcript variant X10 | 1778 | 0.689 | 0.014386049 | 1813 | 0.728 | 0.014189739 | 1446 | 0.729 | 0.013227508 |
| rna4485 | XM_013328317.1 | 5164520 | 5213751 | casein kinase I isoform gamma-3%2C transcript variant X13 | 1778 | 0.689 | 0.014386049 | 1813 | 0.728 | 0.014189739 | 1446 | 0.729 | 0.013227508 |
| rna4486 | XM_013328318.1 | 5164520 | 5213751 | casein kinase I isoform gamma-3%2C transcript variant X14 | 1778 | 0.689 | 0.014386049 | 1813 | 0.728 | 0.014189739 | 1446 | 0.729 | 0.013227508 |
| rna4484 | XM_013328319.1 | 5164520 | 5213751 | casein kinase I isoform gamma-3%2C transcript variant X15 | 1778 | 0.689 | 0.014386049 | 1813 | 0.728 | 0.014189739 | 1446 | 0.729 | 0.013227508 |
| rna4483 | XM_013328320.1 | 5164520 | 5213751 | casein kinase I isoform gamma-3%2C transcript variant X16 | 1778 | 0.689 | 0.014386049 | 1813 | 0.728 | 0.014189739 | 1446 | 0.729 | 0.013227508 |
| rna4487 | XM_013328303.1 | 5164520 | 5213739 | casein kinase I isoform gamma-3%2C transcript variant X1 | 1778 | 0.689 | 0.014391142 | 1813 | 0.728 | 0.014194494 | 1446 | 0.729 | 0.013231931 |
| rna4488 | XM_013328308.1 | 5164520 | 5213731 | casein kinase I isoform gamma-3%2C transcript variant X5 | 1778 | 0.689 | 0.014394538 | 1813 | 0.727 | 0.014197666 | 1446 | 0.729 | 0.013234881 |
| rna4363 | XM_013328344.1 | 1831299 | 1842059 | probable isocitrate dehydrogenase [NAD] subunit alpha%2C mitochondrial%2C transcript variant X2 | 354 | 0.66 | 0.01500007 | 366 | 0.705 | 0.014674234 | 315 | 0.7 | 0.013238679 |
| rna4489 | XM_013328315.1 | 5164520 | 5213712 | casein kinase I isoform gamma-3%2C transcript variant X11 | 1778 | 0.689 | 0.014402613 | 1813 | 0.727 | 0.014205205 | 1446 | 0.729 | 0.013241893 |
| rna4490 | XM_013328307.1 | 5164520 | 5213697 | casein kinase I isoform gamma-3%2C transcript variant X4 | 1778 | 0.688 | 0.014408993 | 1813 | 0.727 | 0.014211163 | 1446 | 0.729 | 0.013247434 |
| rna4491 | XM_013328316.1 | 5164520 | 5213680 | casein kinase I isoform gamma-3%2C transcript variant X12 | 1778 | 0.688 | 0.014416232 | 1813 | 0.727 | 0.014217921 | 1446 | 0.729 | 0.013253719 |
| rna4351 | XM_013328301.1 | 1621486 | 1629516 | monocarboxylate transporter 1-like%2C transcript variant X2 | 253 | 0.677 | 0.015556926 | 240 | 0.64 | 0.015965943 | 224 | 0.676 | 0.013263462 |
| rna4397 | XM_013328166.1 | 2628373 | 2634398 | putative odorant receptor 85d | 171 | 0.636 | 0.014448566 | 180 | 0.667 | 0.014342764 | 151 | 0.625 | 0.013279379 |
| rna4402 | XM_013328221.1 | 2922570 | 2976883 | dynein heavy chain%2C cytoplasmic | 1839 | 0.608 | 0.015209379 | 1734 | 0.624 | 0.013681591 | 1319 | 0.61 | 0.013491432 |
| rna4417 | XM_013328283.1 | 3128643 | 3157699 | phospholipid-transporting ATPase ID | 550 | 0.61 | 0.006533429 | 479 | 0.63 | 0.005536496 | 591 | 0.605 | 0.013728037 |
| rna4367 | XM_013328394.1 | 1871706 | 1877740 | uncharacterized family 31 glucosidase KIAA1161-like%2C transcript variant X1 | 227 | 0.771 | 0.014574838 | 206 | 0.719 | 0.01482246 | 173 | 0.669 | 0.013877593 |
| rna4366 | XM_013328395.1 | 1871706 | 1878974 | uncharacterized family 31 glucosidase KIAA1161-like%2C transcript variant X2 | 227 | 0.64 | 0.014574838 | 206 | 0.597 | 0.01482246 | 173 | 0.556 | 0.013877593 |
| rna4369 | XM_013328416.1 | 1888912 | 1894807 | uncharacterized family 31 glucosidase KIAA1161-like | 187 | 0.646 | 0.015901968 | 190 | 0.675 | 0.01622541 | 165 | 0.618 | 0.013886772 |
| rna4516 | XM_013328385.1 | 5717817 | 5721577 | 40S ribosomal protein S6 | 141 | 0.808 | 0.016680076 | 126 | 0.747 | 0.015159258 | 119 | 0.771 | 0.013921093 |
| rna4457 | XM_013328363.1 | 4325443 | 4337300 | arginine-glutamic acid dipeptide repeats protein-like | 404 | 0.648 | 0.01402339 | 374 | 0.668 | 0.011080246 | 346 | 0.711 | 0.013923976 |
| rna4466 | XM_013328178.1 | 4834313 | 4841636 | chromatin assembly factor 1 subunit A | 268 | 0.691 | 0.014858696 | 302 | 0.781 | 0.01333414 | 254 | 0.765 | 0.013975477 |
| rna4323 | XM_013328353.1 | 1417895 | 1429459 | zinc finger protein 135-like%2C transcript variant X1 | 434 | 0.639 | 0.020277646 | 455 | 0.625 | 0.020355006 | 255 | 0.553 | 0.014106421 |
| rna4320 | XM_013328400.1 | 1384695 | 1394057 | A-kinase anchor protein 17A%2C transcript variant X1 | 253 | 0.593 | 0.013673796 | 230 | 0.567 | 0.014024146 | 220 | 0.588 | 0.014323947 |
| rna4319 | XM_013328401.1 | 1384695 | 1394057 | A-kinase anchor protein 17A%2C transcript variant X2 | 253 | 0.593 | 0.013673796 | 230 | 0.567 | 0.014024146 | 220 | 0.588 | 0.014323947 |
| rna4318 | XM_013328402.1 | 1384695 | 1394057 | A-kinase anchor protein 17A%2C transcript variant X3 | 253 | 0.593 | 0.013673796 | 230 | 0.567 | 0.014024146 | 220 | 0.588 | 0.014323947 |
| rna4317 | XM_013328403.1 | 1384695 | 1394057 | A-kinase anchor protein 17A%2C transcript variant X4 | 253 | 0.593 | 0.013673796 | 230 | 0.567 | 0.014024146 | 220 | 0.588 | 0.014323947 |
| rna4316 | XM_013328404.1 | 1384695 | 1394057 | A-kinase anchor protein 17A%2C transcript variant X5 | 253 | 0.593 | 0.013673796 | 230 | 0.567 | 0.014024146 | 220 | 0.588 | 0.014323947 |
| rna4515 | XM_013328424.1 | 5705067 | 5712195 | geranylgeranyl transferase type-1 subunit beta%2C transcript variant X1 | 319 | 0.777 | 0.019859861 | 354 | 0.801 | 0.020228957 | 302 | 0.816 | 0.014330747 |
| rna4514 | XM_013328425.1 | 5705067 | 5712195 | geranylgeranyl transferase type-1 subunit beta%2C transcript variant X2 | 319 | 0.777 | 0.019859861 | 354 | 0.801 | 0.020228957 | 302 | 0.816 | 0.014330747 |
| rna4341 | XM_013328157.1 | 1524364 | 1540086 | integral membrane protein DGCR2/IDD-like | 500 | 0.627 | 0.016272457 | 506 | 0.645 | 0.015666339 | 380 | 0.617 | 0.014409885 |
| rna4305 | XM_013328265.1 | 917570 | 930179 | uncharacterized LOC106129656 | 383 | 0.516 | 0.017309033 | 347 | 0.477 | 0.017272682 | 344 | 0.577 | 0.014794716 |
| rna4356 | XM_013328227.1 | 1667064 | 1770730 | gonadotropin-releasing hormone II receptor-like | 2501 | 0.502 | 0.013139661 | 2641 | 0.517 | 0.013387471 | 2322 | 0.517 | 0.014889395 |
| rna4326 | XM_013328321.1 | 1449157 | 1465233 | probable ATP-dependent RNA helicase YTHDC2 | 395 | 0.492 | 0.017093656 | 402 | 0.486 | 0.016996883 | 324 | 0.51 | 0.015137028 |
| rna4518 | XM_013328199.1 | 5731508 | 5738487 | ubiquitin-like modifier-activating enzyme ATG7 | 341 | 0.744 | 0.018723624 | 391 | 0.784 | 0.020415932 | 323 | 0.786 | 0.015780398 |
| rna4399 | XM_013328195.1 | 2740022 | 2773970 | hemicentin-2-like | 1411 | 0.7 | 0.018305714 | 1269 | 0.711 | 0.01579168 | 1105 | 0.681 | 0.015959163 |
| rna4388 | XM_013328405.1 | 2562109 | 2571267 | regulator of nonsense transcripts 1 homolog | 260 | 0.623 | 0.01448283 | 234 | 0.63 | 0.011783985 | 212 | 0.593 | 0.016005798 |
| rna4375 | XM_013328207.1 | 2213901 | 2250952 | cell adhesion molecule 3-like | 1117 | 0.553 | 0.017373042 | 1189 | 0.582 | 0.017199421 | 1037 | 0.565 | 0.016310751 |
| rna4335 | XM_013328275.1 | 1490555 | 1505353 | lon protease homolog%2C mitochondrial%2C transcript variant X1 | 441 | 0.495 | 0.019835248 | 460 | 0.506 | 0.018761867 | 353 | 0.514 | 0.016329611 |
| rna4336 | XM_013328276.1 | 1490555 | 1505353 | lon protease homolog%2C mitochondrial%2C transcript variant X2 | 441 | 0.495 | 0.019835248 | 460 | 0.506 | 0.018761867 | 353 | 0.514 | 0.016329611 |
| rna4527 | XM_013328272.1 | 5788149 | 5817440 | synaptic vesicle glycoprotein 2C-like%2C transcript variant X1 | 1090 | 0.654 | 0.016902493 | 1121 | 0.652 | 0.01678627 | 1069 | 0.663 | 0.016341617 |
| rna4364 | XM_013328430.1 | 1849499 | 1858817 | nucleolar protein 6 | 387 | 0.631 | 0.01841051 | 373 | 0.676 | 0.017427999 | 315 | 0.666 | 0.016504625 |
| rna4519 | XM_013328211.1 | 5739062 | 5743761 | 39S ribosomal protein L2%2C mitochondrial%2C transcript variant X2 | 144 | 0.41 | 0.023212282 | 155 | 0.473 | 0.023363934 | 123 | 0.462 | 0.01668886 |
| rna4462 | XM_013328175.1 | 4535269 | 4543692 | elongation factor Tu GTP-binding domain-containing protein 1 | 296 | 0.568 | 0.017343452 | 274 | 0.57 | 0.015638323 | 301 | 0.665 | 0.016740826 |
| rna4346 | XM_013328159.1 | 1568083 | 1573750 | katanin p60 ATPase-containing subunit A-like 2 | 253 | 0.786 | 0.017294694 | 244 | 0.793 | 0.015920924 | 229 | 0.799 | 0.016830798 |
| rna4520 | XM_013328210.1 | 5739062 | 5742744 | 39S ribosomal protein L2%2C mitochondrial%2C transcript variant X1 | 138 | 0.493 | 0.02372714 | 148 | 0.568 | 0.023889944 | 119 | 0.568 | 0.016839515 |
| rna4465 | XM_013328384.1 | 4817352 | 4831584 | uncharacterized LOC106129727 | 595 | 0.638 | 0.018827495 | 612 | 0.672 | 0.017066858 | 481 | 0.619 | 0.016952938 |
| rna4311 | XM_013328154.1 | 1063427 | 1075258 | uncharacterized LOC106129557 | 549 | 0.639 | 0.021962126 | 500 | 0.634 | 0.020626919 | 461 | 0.699 | 0.016967 |
| rna4474 | XM_013328347.1 | 5144998 | 5163317 | uncharacterized LOC106129699%2C transcript variant X1 | 851 | 0.696 | 0.018764671 | 789 | 0.691 | 0.017536698 | 694 | 0.709 | 0.017073215 |
| rna4475 | XM_013328349.1 | 5145472 | 5163317 | uncharacterized LOC106129699%2C transcript variant X2 | 839 | 0.688 | 0.019198705 | 776 | 0.686 | 0.017860861 | 685 | 0.701 | 0.017474922 |
| rna4289 | XM_013328208.1 | 507747 | 509855 | uncharacterized LOC106129607 | 108 | 0.786 | 0.019687015 | 90 | 0.794 | 0.017459236 | 108 | 0.843 | 0.017548592 |
| rna4463 | XM_013328176.1 | 4664744 | 4714572 | hemicentin-2-like | 2152 | 0.606 | 0.020292481 | 2118 | 0.62 | 0.018615133 | 1829 | 0.63 | 0.017549343 |
| rna4458 | XM_013328284.1 | 4354138 | 4400298 | uncharacterized protein YHR080C-like%2C transcript variant X1 | 1614 | 0.56 | 0.016665457 | 1534 | 0.571 | 0.013892726 | 1408 | 0.586 | 0.017572023 |
| rna4358 | XM_013328356.1 | 1811917 | 1826990 | phosphatidylinositol 3-kinase catalytic subunit type 3 | 608 | 0.589 | 0.022799511 | 628 | 0.608 | 0.022059835 | 474 | 0.564 | 0.017755397 |
| rna4470 | XM_013328294.1 | 4961729 | 5141211 | furin-like protease 1%2C isoform 1-CRR%2C transcript variant X1 | 6652 | 0.56 | 0.018331297 | 6785 | 0.572 | 0.018259894 | 5726 | 0.587 | 0.01777704 |
| rna4471 | XM_013328295.1 | 4961729 | 5141211 | furin-like protease 1%2C isoform 1-CRR%2C transcript variant X2 | 6652 | 0.56 | 0.018331297 | 6785 | 0.572 | 0.018259894 | 5726 | 0.587 | 0.01777704 |
| rna4468 | XM_013328297.1 | 4961729 | 5141211 | furin-like protease 1%2C isoform 1-CRR%2C transcript variant X4 | 6652 | 0.56 | 0.018331297 | 6785 | 0.572 | 0.018259894 | 5726 | 0.587 | 0.01777704 |
| rna4469 | XM_013328299.1 | 4961729 | 5141211 | furin-like protease 1%2C isoform 1-CRR%2C transcript variant X6 | 6652 | 0.56 | 0.018331297 | 6785 | 0.572 | 0.018259894 | 5726 | 0.587 | 0.01777704 |
| rna4353 | XM_013328365.1 | 1635267 | 1650031 | AP-3 complex subunit beta-2 | 497 | 0.482 | 0.019673635 | 517 | 0.493 | 0.019788226 | 408 | 0.484 | 0.01778626 |
| rna4473 | XM_013328296.1 | 4961729 | 5139591 | furin-like protease 1%2C isoform 1-CRR%2C transcript variant X3 | 6605 | 0.558 | 0.018454357 | 6735 | 0.57 | 0.018383854 | 5691 | 0.585 | 0.017901082 |
| rna4472 | XM_013328298.1 | 4961729 | 5139591 | furin-like protease 1%2C isoform 1-CRR%2C transcript variant X5 | 6605 | 0.558 | 0.018454357 | 6735 | 0.57 | 0.018383854 | 5691 | 0.585 | 0.017901082 |
| rna4334 | XM_013328156.1 | 1485721 | 1490391 | probable tRNA (guanine(26)-N(2))-dimethyltransferase | 215 | 0.821 | 0.018499936 | 223 | 0.84 | 0.017830596 | 172 | 0.79 | 0.017903263 |
| rna4459 | XM_013328286.1 | 4367881 | 4400298 | uncharacterized protein YHR080C-like%2C transcript variant X2 | 1022 | 0.514 | 0.01637634 | 992 | 0.521 | 0.014090233 | 924 | 0.538 | 0.017914521 |
| rna4379 | XM_013328205.1 | 2401400 | 2408494 | microfibrillar-associated protein 1 | 217 | 0.598 | 0.01490628 | 209 | 0.613 | 0.013053112 | 195 | 0.579 | 0.017974082 |
| rna4528 | XM_013328274.1 | 5788149 | 5807240 | synaptic vesicle glycoprotein 2C-like%2C transcript variant X2 | 781 | 0.667 | 0.018624867 | 796 | 0.654 | 0.018646787 | 777 | 0.679 | 0.017990336 |
| rna4494 | XM_013328340.1 | 5239658 | 5249675 | alpha-tocopherol transfer protein-like | 543 | 0.667 | 0.024193356 | 535 | 0.671 | 0.023512712 | 475 | 0.728 | 0.017992505 |
| rna4377 | XM_013328202.1 | 2397004 | 2399474 | acyl-CoA Delta(11) desaturase-like | 95 | 0.807 | 0.013121725 | 91 | 0.826 | 0.012078733 | 105 | 0.853 | 0.018038584 |
| rna4370 | XM_013328371.1 | 1915714 | 1997644 | gonadotropin-releasing hormone receptor%2C transcript variant X1 | 3020 | 0.565 | 0.017658915 | 3207 | 0.582 | 0.018459627 | 2565 | 0.538 | 0.018231373 |
| rna4371 | XM_013328372.1 | 1915714 | 1997644 | gonadotropin-releasing hormone receptor%2C transcript variant X2 | 3020 | 0.565 | 0.017658915 | 3207 | 0.582 | 0.018459627 | 2565 | 0.538 | 0.018231373 |
| rna4297 | XM_013328212.1 | 878042 | 880103 | 60S ribosomal protein L36 | 74 | 0.586 | 0.017021303 | 66 | 0.605 | 0.014981928 | 98 | 0.756 | 0.018501056 |
| rna4398 | XM_013328251.1 | 2650908 | 2658501 | odorant receptor 46a%2C isoform A-like | 374 | 0.816 | 0.017949239 | 348 | 0.817 | 0.015522419 | 310 | 0.798 | 0.018573135 |
| rna4513 | XM_013328183.1 | 5584108 | 5587497 | zinc metalloproteinase nas-26-like | 170 | 0.727 | 0.020259458 | 191 | 0.693 | 0.022580203 | 156 | 0.725 | 0.018650785 |
| rna4313 | XM_013328155.1 | 1323121 | 1327462 | uncharacterized LOC106129558 | 271 | 0.757 | 0.027620403 | 255 | 0.767 | 0.02655026 | 160 | 0.666 | 0.018674776 |
| rna4283 | XM_013328150.1 | 117071 | 202199 | COUP transcription factor 2 | 3901 | 0.639 | 0.020747332 | 3839 | 0.644 | 0.020308331 | 3463 | 0.656 | 0.018676525 |
| rna4524 | XM_013328407.1 | 5764182 | 5774900 | ejaculatory bulb-specific protein 3-like%2C transcript variant X1 | 424 | 0.663 | 0.017350391 | 448 | 0.695 | 0.016681601 | 457 | 0.695 | 0.018691693 |
| rna4349 | XM_013328160.1 | 1589746 | 1617720 | ras-specific guanine nucleotide-releasing factor 2-like | 1042 | 0.63 | 0.018533524 | 1086 | 0.664 | 0.016560191 | 874 | 0.629 | 0.018795288 |
| rna4299 | XM_013328256.1 | 886270 | 900020 | histone H4 transcription factor%2C transcript variant X1 | 577 | 0.604 | 0.020228768 | 579 | 0.659 | 0.018517783 | 538 | 0.662 | 0.018815606 |
| rna4344 | XM_013328396.1 | 1559700 | 1562822 | membrane-bound alkaline phosphatase-like | 138 | 0.786 | 0.021208463 | 136 | 0.814 | 0.019345777 | 129 | 0.795 | 0.019003645 |
| rna4523 | XM_013328408.1 | 5763005 | 5774900 | ejaculatory bulb-specific protein 3-like%2C transcript variant X2 | 482 | 0.667 | 0.017765032 | 494 | 0.693 | 0.016863614 | 521 | 0.702 | 0.019232355 |
| rna4324 | XM_013328355.1 | 1429485 | 1433468 | D-amino-acid oxidase | 180 | 0.769 | 0.022401047 | 171 | 0.748 | 0.021670132 | 150 | 0.721 | 0.019578476 |
| rna4512 | XM_013328245.1 | 5523782 | 5567906 | lachesin-like | 1999 | 0.614 | 0.022743925 | 2034 | 0.622 | 0.022294619 | 1683 | 0.619 | 0.019783072 |
| rna4533 | XM_013328302.1 | 5936832 | 5986228 | tyrosine-protein phosphatase non-receptor type 9-like | 1915 | 0.593 | 0.018338658 | 1984 | 0.597 | 0.019156148 | 1753 | 0.582 | 0.020455713 |
| rna4365 | XM_013328213.1 | 1859120 | 1867969 | uncharacterized family 31 glucosidase KIAA1161-like | 333 | 0.553 | 0.01865628 | 301 | 0.504 | 0.018463486 | 278 | 0.574 | 0.020574957 |
| rna4325 | XM_013328323.1 | 1434200 | 1447798 | TFIIH basal transcription factor complex helicase XPD subunit | 470 | 0.483 | 0.023278871 | 455 | 0.464 | 0.023244102 | 403 | 0.505 | 0.020644034 |
| rna4312 | XM_013328390.1 | 1276242 | 1283154 | collagenase-like | 281 | 0.542 | 0.023178141 | 278 | 0.537 | 0.023880987 | 249 | 0.578 | 0.020653982 |
| rna4355 | XM_013328209.1 | 1658562 | 1661332 | gbkey=mRNA | 132 | 0.851 | 0.016475081 | 118 | 0.766 | 0.016984606 | 109 | 0.725 | 0.02076845 |
| rna4368 | XM_013328190.1 | 1881341 | 1887617 | uncharacterized family 31 glucosidase KIAA1161-like | 271 | 0.578 | 0.019633061 | 263 | 0.578 | 0.019645999 | 252 | 0.593 | 0.020841519 |
| rna4314 | XM_013328230.1 | 1353928 | 1366053 | cytochrome P450 6B2-like | 506 | 0.545 | 0.025493476 | 436 | 0.532 | 0.021572619 | 386 | 0.52 | 0.020849574 |
| rna4307 | XM_013328263.1 | 932579 | 937549 | survival motor neuron protein%2C transcript variant X1 | 268 | 0.717 | 0.023064884 | 286 | 0.677 | 0.024100881 | 210 | 0.621 | 0.021044792 |
| rna4306 | XM_013328264.1 | 932579 | 937549 | survival motor neuron protein%2C transcript variant X2 | 268 | 0.717 | 0.023064884 | 286 | 0.677 | 0.024100881 | 210 | 0.621 | 0.021044792 |
| rna4308 | XM_013328413.1 | 938212 | 946286 | uncharacterized LOC106129749 | 448 | 0.713 | 0.023860812 | 489 | 0.762 | 0.024650573 | 347 | 0.713 | 0.021213124 |
| rna4495 | XM_013328337.1 | 5249756 | 5270691 | alpha-tocopherol transfer protein-like | 1070 | 0.64 | 0.023430361 | 1089 | 0.665 | 0.021801526 | 932 | 0.655 | 0.021302354 |
| rna4532 | XM_013328391.1 | 5891606 | 5936552 | bumetanide-sensitive sodium-(potassium)-chloride cotransporter | 1427 | 0.474 | 0.021341498 | 1417 | 0.459 | 0.021724603 | 1381 | 0.472 | 0.021385654 |
| rna4499 | XM_013328329.1 | 5273552 | 5310024 | DENN domain-containing protein 4C%2C transcript variant X1 | 1542 | 0.563 | 0.022557767 | 1455 | 0.543 | 0.021318098 | 1466 | 0.579 | 0.0219706 |
| rna4497 | XM_013328330.1 | 5273552 | 5310024 | DENN domain-containing protein 4C%2C transcript variant X2 | 1542 | 0.563 | 0.022557767 | 1455 | 0.543 | 0.021318098 | 1466 | 0.579 | 0.0219706 |
| rna4498 | XM_013328332.1 | 5273552 | 5310024 | DENN domain-containing protein 4C%2C transcript variant X3 | 1542 | 0.563 | 0.022557767 | 1455 | 0.543 | 0.021318098 | 1466 | 0.579 | 0.0219706 |
| rna4401 | XM_013328254.1 | 2892093 | 2895881 | uncharacterized LOC106129649 | 177 | 0.741 | 0.018112111 | 173 | 0.774 | 0.01880833 | 148 | 0.73 | 0.02242318 |
| rna4286 | XM_013328255.1 | 296812 | 298122 | uncharacterized LOC106129650 | 75 | 0.73 | 0.024917858 | 71 | 0.683 | 0.026239023 | 60 | 0.686 | 0.023446898 |
| rna4292 | XM_013328420.1 | 782839 | 797582 | organic cation transporter protein-like | 940 | 0.76 | 0.024639982 | 874 | 0.727 | 0.024891206 | 791 | 0.746 | 0.023580162 |
| rna4295 | XM_013328191.1 | 851105 | 875577 | organic cation transporter protein-like%2C transcript variant X1 | 1225 | 0.591 | 0.024989271 | 1111 | 0.58 | 0.023653437 | 1056 | 0.607 | 0.023840622 |
| rna4296 | XM_013328194.1 | 852790 | 875577 | organic cation transporter protein-like%2C transcript variant X2 | 1131 | 0.579 | 0.024994831 | 1019 | 0.567 | 0.023516426 | 975 | 0.595 | 0.023936719 |
| rna4301 | XM_013328257.1 | 892898 | 900020 | histone H4 transcription factor%2C transcript variant X2 | 437 | 0.702 | 0.026294065 | 427 | 0.694 | 0.026057796 | 405 | 0.716 | 0.024658554 |
| rna4464 | XM_013328383.1 | 4810659 | 4814482 | peptidyl-prolyl cis-trans isomerase G | 204 | 0.703 | 0.022109041 | 215 | 0.754 | 0.021423466 | 147 | 0.554 | 0.024671476 |
| rna4504 | XM_013328334.1 | 5452362 | 5457029 | pancreatic lipase-related protein 2-like | 314 | 0.79 | 0.023964422 | 329 | 0.781 | 0.024215162 | 275 | 0.771 | 0.025397708 |
| rna4502 | XM_013328397.1 | 5438263 | 5441330 | pancreatic lipase-related protein 2-like | 178 | 0.691 | 0.028531789 | 175 | 0.694 | 0.02718466 | 160 | 0.666 | 0.025959637 |
| rna4293 | XM_013328374.1 | 808877 | 830079 | organic cation transporter protein-like%2C transcript variant X1 | 1004 | 0.551 | 0.026870029 | 967 | 0.537 | 0.025994914 | 886 | 0.537 | 0.026360269 |
| rna4291 | XM_013328431.1 | 633693 | 760199 | lachesin-like | 5027 | 0.438 | 0.026772375 | 4845 | 0.431 | 0.026513013 | 4484 | 0.433 | 0.026460228 |
| rna4503 | XM_013328179.1 | 5446800 | 5451715 | pancreatic lipase-related protein 2-like | 299 | 0.699 | 0.030131834 | 314 | 0.727 | 0.03010721 | 296 | 0.736 | 0.026774886 |
| rna4287 | XM_013328252.1 | 328410 | 329562 | uncharacterized LOC106129647 | 69 | 0.635 | 0.030412102 | 66 | 0.547 | 0.03066087 | 67 | 0.714 | 0.026841506 |
| rna4294 | XM_013328375.1 | 808877 | 828008 | organic cation transporter protein-like%2C transcript variant X2 | 855 | 0.518 | 0.027457545 | 839 | 0.508 | 0.02640907 | 759 | 0.5 | 0.026858788 |
| rna4280 | XM_013328223.1 | 37998 | 40521 | proteasome subunit beta type-3 | 166 | 0.697 | 0.030671404 | 145 | 0.657 | 0.028231876 | 161 | 0.736 | 0.027159099 |
| rna4290 | XM_013328153.1 | 518291 | 555617 | limbic system-associated membrane protein-like | 2167 | 0.628 | 0.027881285 | 2052 | 0.605 | 0.02781822 | 2100 | 0.633 | 0.027433434 |
| rna4410 | XM_013328222.1 | 3055117 | 3067241 | cyclin-dependent kinase 7 | 123 | 0.462 | 0.004064722 | 90 | 0.451 | 0.003204948 | 122 | 0.388 | na |
| rna4278 | XM_013328389.1 | 2546 | 14627 | gbkey=mRNA | 119 | 0.179 | na | 95 | 0.153 | na | 161 | 0.285 | na |
| rna4284 | XM_013328151.1 | 264305 | 268869 | uncharacterized LOC106129553 | 48 | 0.207 | na | 54 | 0.212 | na | 74 | 0.229 | na |
| rna4285 | XM_013328289.1 | 269265 | 295932 | mediator of DNA damage checkpoint protein 1-like | 645 | 0.347 | na | 596 | 0.355 | na | 635 | 0.396 | na |
| rna4288 | XM_013328152.1 | 348091 | 349107 | uncharacterized LOC106129555 | 0 | 0 | na | 0 | 0 | na | 0 | 0 | na |
| rna4304 | XM_013328259.1 | 900124 | 912396 | spermine oxidase-like%2C transcript variant X1 | 208 | 0.223 | na | 191 | 0.207 | na | 199 | 0.229 | na |
| rna4302 | XM_013328261.1 | 900124 | 920223 | spermine oxidase-like%2C transcript variant X2 | 522 | 0.388 | na | 487 | 0.371 | na | 442 | 0.396 | na |
| rna4303 | XM_013328262.1 | 900124 | 913399 | spermine oxidase-like%2C transcript variant X3 | 208 | 0.206 | na | 191 | 0.191 | na | 199 | 0.212 | na |
| rna4309 | XM_013328218.1 | 949659 | 955857 | uncharacterized LOC106129616 | 25 | 0.042 | na | 21 | 0.058 | na | 26 | 0.061 | na |
| rna4315 | XM_013328406.1 | 1367764 | 1382026 | chymotrypsin BII-like | 288 | 0.246 | na | 237 | 0.219 | na | 198 | 0.219 | na |
| rna4327 | XM_013328324.1 | 1462726 | 1464554 | uncharacterized LOC106129685 | 3 | 0.04 | na | 5 | 0.021 | na | 0 | 0 | na |
| rna4373 | XM_013328373.1 | 1953448 | 1954299 | uncharacterized LOC106129718 | 0 | 0 | na | 0 | 0 | na | 0 | 0 | na |
| rna4374 | XM_013328162.1 | 2097656 | 2098856 | uncharacterized LOC106129567 | 13 | 0.06 | na | 13 | 0.062 | na | 14 | 0.062 | na |
| rna4376 | XM_013328164.1 | 2257330 | 2262771 | uncharacterized LOC106129568 | 12 | 0.071 | na | 13 | 0.064 | na | 7 | 0.044 | na |
| rna4404 | XM_013328220.1 | 2996162 | 2999913 | polyubiquitin-B | 58 | 0.353 | na | 56 | 0.37 | na | 49 | 0.369 | na |
| rna4412 | XM_013328359.1 | 3079983 | 3096923 | ubiquilin-1-like | 132 | 0.243 | na | 133 | 0.259 | na | 156 | 0.245 | na |
| rna4435 | XM_013328268.1 | 3476653 | 3481076 | uncharacterized LOC106129658 | 0 | 0.009 | na | 0 | 0.004 | na | 0 | 0.009 | na |
| rna4437 | XM_013328266.1 | 3479317 | 3481542 | uncharacterized LOC106129657%2C transcript variant X1 | 0 | 0 | na | 0 | 0 | na | 0 | 0 | na |
| rna4436 | XM_013328267.1 | 3479317 | 3481542 | uncharacterized LOC106129657%2C transcript variant X2 | 0 | 0 | na | 0 | 0 | na | 0 | 0 | na |
| rna4492 | XM_013328338.1 | 5215501 | 5234703 | alpha-tocopherol transfer protein-like | 558 | 0.365 | na | 593 | 0.387 | na | 400 | 0.356 | na |
| rna4505 | XM_013328335.1 | 5457125 | 5461303 | pancreatic lipase-related protein 2-like | 91 | 0.351 | na | 93 | 0.343 | na | 84 | 0.369 | na |
