# Supplementary material for: Selective Sweeps in a Nutshell: The Genomic Footprint of Rapid Insecticide Resistance Evolution in the Almond Agroecosystem
Source: Genome Biol Evol. 2020 Nov 4;13(1):evaa234. doi: 10.1093/gbe/evaa234 (PMC7850051; doi:10.1093/gbe/evaa234)
Supplement: evaa234_Supplementary_Data [file evaa234_supplementary_data.zip › Table S5.docx]

**Table S5.** The twenty most differentiated SNPs between the reference genome (SPIRL-1966), ALM, FIG and R using Mahalanobis distances (PCAdapt, 1.1, Luu et al., 2017). **po**s: position along scaffold, **rc**: reference character, **allele count:** number of alleles found in all populations; **allele states**: allele characters in all populations (sorted by counts in all populations); **del sum**: sum of deletions in all populations (should be zero, if not the postion may not be reliable); **snp type**: SNP type: “pop” a SNP within or between the populations; “rc” a SNP between the reference sequence character and the consensus of at least one populaton; “rc|pop” both; **major alleles**: most frequent allele in all populations [ALM FIG R]; **minor alleles**: second most frequent allele in all populations [ALM FIG R]

| **Scaffold#** | **pos** | **Ref** | **allele count** | **allele states** | **SNP type** | **major alleles (maa)** | **minor alleles (mia)** | **FDR pval.** | **gene model functional annotation** |
| --- | --- | --- | --- | --- | --- | --- | --- | --- | --- |
| NW_013535379.1 | 775604 | A | 3 | A/C/G | pop | AAA | CCC | 0.01859 | inter-genic, nearby CYP341 cluster of P450s |
| NW_013535334.1 | 84304 | G | 3 | G/T/A | pop | GGG | TTT | 0.01970 | zinc finger 224-like |
| NW_013535359.1 | 304369 | A | 2 | A/T | pop | AAA | TTT | 0.01970 | inter-genic |
| NW_013535509.1 | 95606 | C | 2 | C/A | rc\|pop | ACC | CAA | 0.01970 | inter-genic just upstream of Ecdisteroid Kinase cluster |
| NW_013535530.1 | 170651 | T | 2 | T/C | pop | TTT | CCC | 0.01970 | inter-genic |
| NW_013535492.1 | 2659949 | T | 3 | A/G/T | rc\|pop | AAA | TGG | 0.03339 | intronic mannose-1-phosphate guanyltransferase alpha |
| NW_013535509.1 | 185180 | T | 2 | T/A | rc\|pop | TTA | AAT | 0.03339 | intergenic in Ecdisyteroid kinase gene cluster |
| NW_013535854.1 | 148606 | C | 2 | C/T | pop | CCC | TTT | 0.03339 | intergenic, just downstream of sorting nexin-2 |
| NW_013535700.1 | 310754 | A | 2 | A/G | pop | AAA | GGG | 0.04845 | intergenic, just downstream of protein king tubby |
| NW_013535323.1 | 5604262 | T | 2 | T/G | pop | TTT | GGG | 0.05023 | intergenic |
| NW_013535492.1 | 2832430 | G | 2 | G/C | rc\|pop | GCG | CGC | 0.05023 | intronic protein CIP2A homolog |
| NW_013535386.1 | 3083373 | G | 2 | G/A | pop | GGG | AAA | 0.05948 | intronic teneurin-3 (downstream of CYP4G cluster) |
| NW_013535423.1 | 1590364 | A | 2 | A/C | pop | AAA | CCC | 0.05948 | intronic neuropeptide F-like |
| NW_013535319.1 | 2357306 | G | 2 | G/A | pop | GGG | AAA | 0.06952 | intronic nuclear migration protein nudC |
| NW_013535323.1 | 7237902 | T | 2 | T/A | pop | TTT | AAA | 0.06952 | intergenic |
| NW_013535332.1 | 4322388 | A | 2 | A/T | pop | AAA | TTT | 0.06952 | intronic protein yellow-like |
| NW_013535362.1 | 4099150 | T | 2 | T/C | rc\|pop | CCT | TTC | 0.06952 | at intron-exon boundary uncharacterized LOC10612973 |
| NW_013535378.1 | 1393893 | T | 2 | T/C | pop | TTT | CCC | 0.06952 | intergenic |
| NW_013535378.1 | 874667 | T | 2 | T/C | pop | TTT | CCC | 0.06952 | intergenic |
