# Supplementary material for: Selective Sweeps in a Nutshell: The Genomic Footprint of Rapid Insecticide Resistance Evolution in the Almond Agroecosystem
Source: Genome Biol Evol. 2020 Nov 4;13(1):evaa234. doi: 10.1093/gbe/evaa234 (PMC7850051; doi:10.1093/gbe/evaa234)
Supplement: evaa234_Supplementary_Data [file evaa234_supplementary_data.zip › Table S7.docx]

**Table S7.** Bifenthrin use as the active ingredient(s) under registered trade names from 2006 – 2017 in almond orchards. Usage intensity (*UI*) is equal to the pounds of bifenthrin applied for each product divided by the treated acres. Trade names which comprise “Other” include Bifenture^®^ EC-CA, Capture^®^ EC-Cal, Swagger^®^, Helena Bifenthrin^®^ 2EC-Cal, Sniper^®^ Helios, Bifen 2 Ag Gold-Cal, Brigade^®^ 2EC, Fanfare® EC, SPECKoZ^®^ Bifenthrin, Bifenture^®^ LFC, and Bifen 25% EC.
